# MicroRNA gene dynamics in immune cell subpopulations during aging and atherosclerosis disease development at single-cell resolution

**DOI:** 10.1186/s13073-025-01530-9

**Published:** 2025-10-06

**Authors:** Ana Hernández de Sande, Tanja Turunen, Maria Bouvy-Liivrand, Tiit Örd, Senthil Palani, Mari Lahnalampi, Celia Tundidor-Centeno, Heidi Liljenbäck, Jenni Virta, Henri Niskanen, Buddika Jayasingha, Olli-Pekka Smålander, Lasse Sinkkonen, Lea Mikkola, Thomas Sauter, Anne Roivainen, Tapio Lönnberg, Minna U. Kaikkonen, Merja Heinäniemi

**Affiliations:** 1 School of Medicine, University of Eastern Finland, 70200 Kuopio, North-Savo Finland; 2 Department of Chemistry and Biotechnology, Tallinn University of Technology, 12616 Tallinn, Estonia; 3 A. I. Virtanen Institute, University of Eastern Finland, 70200 Kuopio, North-Savo Finland; 4 Turku PET Centre, University of Turkuand, Turku University Hospital , 20520 Turku, Finland; 5 Turku Center for Disease Modeling, University of Turku, 20520 Turku, Finland; 6 Department of Neurology, Helsinki University Hospital, Helsinki, Finland; 7 Department of Life Science and Medicine (DLSM), University of Luxembourg, 4362 Belvaux, Luxembourg; 8 InFLAMES Research Flagship Center, University of Turku, 20520 Turku, Finland; 9 Turku Bioscience Centre, University of Turku and Åbo Akademi University, 20520 Turku, Finland

**Keywords:** MiRNA genes, Single-cell transcriptomics, Immune cells, Aging, Atherosclerosis, High-fat diet

## Abstract

**Background:**

Regulatory networks controlling aging and disease trajectories remain incompletely understood. MicroRNAs (miRNAs) are a class of regulatory non-coding RNAs that contribute to the regulation of tissue homeostasis by modulating the stability and abundance of their target mRNAs. MiRNA genes are transcribed similarly to protein-coding genes which has facilitated their annotation and quantification from bulk transcriptomes. Here, we show that droplet, spatial, and plate-based single-cell RNA-sequencing platforms can be used to decipher miRNA gene signatures at cellular resolution to reveal their expression dynamics in vivo.

**Methods:**

We first benchmarked the approach examining concordance between platforms, species, and cell type-specific bulk expression data. To discover changes in miRNA gene expression that could contribute to the progressive loss of cellular homeostasis during aging and disease development, we annotated the comprehensive aging mouse dataset, Tabula Muris Senis, with cell type-specific miRNA expression and acquired transcriptome and translatome profiles from an atherosclerosis disease model.

**Results:**

We generated an openly available workflow and aging-profile resource to characterize miRNA expression from single-cell genomics studies. Comparing immune cells in spleen tissue between young and old mice revealed concordance with previous functional studies, highlighting the upregulation of mmu-mir-146a, mmu-mir-101a, and mmu-mir-30 family genes involved in senescence and inflammatory pathways. Atherosclerosis progression is reflected within adipose tissue as expansion of the myeloid compartment, with elevated pro-inflammatory mmu-mir-511 expression in several macrophage subtypes. Upregulation of the immunosuppressive mmu-mir-23b ~ mir-24–2 ~ mir-27b locus was specific to Trem2 + lipid-associated macrophages, prevalent at late disease. Accordingly, ribosome-associated RNA profiling from myeloid cells in vivo validated significant mmu-mir-23b target gene enrichment in disease-regulated translatomes. Prominent tissue infiltration of monocytes led to upregulated mmu-mir-1938 and mmu-mir-22 expression and in classical monocytes activated mmu-mir-221 ~ 222, mmu-mir-511, and mmu-mir-155 gene loci, confirmed by bulk nascent transcriptomics data from ex vivo macrophage cultures. Overall, the monocyte-associated changes in miRNA expression represented the most significant target gene associations in the disease-trajectory translatome profiles.

**Conclusions:**

We demonstrate that miRNA gene transcriptional activity is widely impacted in immune cells by aging and during disease development and further identify the corresponding translatome signature of inflamed adipose tissue.

**Supplementary Information:**

The online version contains supplementary material available at 10.1186/s13073-025-01530-9.

## Background

The study of single-cell transcriptomes has revolutionized the field of cell biology, enabling the identification of new cell types, cellular states, and characterizing cellular transitions across healthy tissues and during disease development [1]. MicroRNAs (miRNAs), a class of regulatory non-coding RNA molecules, can base pair to their target messenger RNA (mRNA), thereby interfering with their translation into proteins. Thus, miRNA-mediated post-transcriptional regulation strongly impacts gene regulatory networks that modulate cell function via controlling cell homeostasis [2]. At the systems level, control of homeostasis and cell state transitions deteriorates over time, impairing tissue function during aging. This process manifests with low-grade tissue inflammation and constitutes a risk for developing inflammatory-related diseases such as diabetes, atherosclerosis, Alzheimer’s disease, and certain cancers (reviewed in [3, 4]).

Recently, new insight into the aging and disease processes has been provided through single-cell RNA-sequencing (scRNA-seq) technologies that enable comprehensive analysis of gene expression changes at cellular resolution. MiRNA genes correspond to long transcripts called primary miRNAs (pri-miRNA) transcribed by RNA polymerase II similar to protein-coding genes. Subsequently, miRNA transcripts are processed into short transcripts, pre-miRNAs, and further into 20–22 nucleotides (nt) long mature miRNAs. In standard bulk RNA sequencing, the size selection step excludes the processed miRNA transcripts. Therefore, separate protocols for small RNA sequencing were developed and represent the most common miRNA profiling method (reviewed in [5]). However, the feasibility of sc-small RNA-seq is limited by low throughput [6, 7]. Acknowledging that single-cell transcriptomics captures between 17 and 23% of unspliced reads [8], analysis of pri-miRNA transcripts presents an alternative. In our previous work, we developed a comprehensive miRNA gene annotation approach based on nascent transcriptome (Global-run-on coupled with sequencing, GRO-seq), Cap Analysis of Gene Expression (CAGE), and histone marker data that enabled the quantification of pri-miRNA transcriptional activity in a multitude of bulk genomics studies in cell lines and primary tissue contexts [9, 10]. Presently, comprehensive gene annotations such as RefSeq [11] that are commonly utilized in single-cell studies primarily consist of pre-miRNA coordinates, appropriate for small RNA sequencing data analysis rather than quantification of miRNA genes. Consequently, the analysis of their regulation at cell-type resolution is lacking, leaving our understanding of regulatory networks that govern cell state homeostasis incomplete.

Here, we leverage our previous approach annotating miRNA gene coordinates to quantify miRNA genes from single-cell transcriptomes and benchmark application to commonly adopted scRNA-seq platforms using the Tabula Muris Senis (TMS) dataset [12–14]. Better understanding of the miRNA post-transcriptional regulatory networks that fine tune cytokine expression and response dynamics upon inflamed state can provide early detection approaches to prevent progression of disease-related functional changes over time. Focusing on the cross-talk between tissue-resident and circulating immune cells, we focused on high-fat diet-induced adipose tissue changes in an atherosclerosis disease model to acquire new cell type-specific transcription and translatome profiles of disease development.

## Methods

### Data collection

We utilized inhouse collected GRO-seq data GSE241550 [15] and Zenodo data [16, 17] and publicly available GRO-seq data (GSE101116 [18, 19], GSE23622 [20, 21], GSE26512 [22, 23], GSE27037 [24, 25], GSE40173 [26, 27], GSE45517 [28, 29], GSE46494 [30, 31], GSE48759 [32, 33], GSE50944 [34, 35], GSE48895 [36, 37], GSE56747 [38, 39], GSE59486 [40, 41]), and FANTOM5 mouse hCAGE datasets [42–46] (cell line, fractionation, primary cell, time course, and tissue data), refer to Additional file 2: Table S1A for sample codes, to define mouse miRNA gene coordinates.

The single-cell datasets from spleen of 3-month-old mice (*n* = 1, male and female) produced by the TMS [12, 13, 47] and from human spleen by Tabula Sapiens [48, 49] consortia (refer to Additional file 2: Table S1B and C for sample codes) together with mature miRNA RT-PCR data collected from mouse immune cells by [50, 51] allowed us to compare how data produced using different single-cell technologies serve in quantifying miRNA gene expression both in mouse and human. Next, we collected 10x Genomics scRNA-seq GSE241567 [52] of the mouse stromal cell line ST2 and compared primary transcription assayed using bulk GRO-seq from the same cell line GSE241550 [15]. Finally, to confirm the compatibility of our approach with spatial technologies, we used 10x Genomics-based spatial platform Slide-tags data [53] collected from mouse E14 hippocampus GSE244355 [54].

To study miRNA gene dynamics during aging, we used the TMS datasets [47] collected using 10x Genomics platform from spleen of 1 month (*n* = 1 male), 3 months (*n* = 1, both male and female), 18 months (*n* = 2 female), 21 months (*n* = 2 female), 24 months (*n* = 2 male), and 30 months (*n* = 4 male) old mice. To study miRNA gene dynamics during disease development, we collected scRNA-seq data GSE241552 [55] from epididymal white adipose tissue (eWAT) and CD115 + blood monocytes of mice representing prelesion, early, inflammatory challenged, and late disease state (*n* = 3 per condition) in an atherosclerosis mouse model (refer to Additional file 2: Table S1B for sample codes).

To validate that the changes detected from scRNA-seq profiles represent regulation of the transcriptional activity at miRNA gene loci, we used GRO-seq profiles collected from two different experimental setups: ex vivo LPS stimulation of bone marrow-derived CD14 + macrophages GSE60857 [56] (referred to as BMDM) and LPS stimulation of peritoneal MPs GSE48759 [33] (referred to as PM, resembling tissue-resident MP) (Additional file 2: Table S1D). To validate that the modulation of miRNA expression can impact target gene mRNA translation, we performed the Translating Ribosome-associated mRNA capture (TRAP)-seq assay with pulldown from WAT Csf1r-expressing myeloid cells (monocytes and macrophages), comparing chow to a 3-month high-fat diet in an atherosclerosis mouse model GSE254396 [57] (*n* = 6 per condition), refer to Additional file 2: Table S1D for sample codes.

Finally, we carried out a measurement of mature miRNA levels at bulk tissue level in WAT, spleen, and isolated blood monocytes using RT-qPCR analysis from matched animals (WAT: *n* = 6 for all conditions except LD *n* = 3, spleen: *n* = 6 per condition, and blood monocytes (data from pool of 3 animals)).

### Annotation of miRNA gene coordinates in the mouse genome

To distinguish primary transcripts corresponding to miRNA genes in mice, we followed the strategy we introduced in [9] and extended in [10]. To define mouse pri-miRNA genes, GRO-seq and CAGE-seq data aligned to the mm9 genome were used to define genomic intervals that correspond to active primary transcription, separated by active TSS (samples used are Listed in Additional file 2: Table S1A and in [9] for mouse and human coordinates, respectively). Due to fewer mouse GRO-seq samples available, the known RefSeq [11] and UCSC known gene (2018) annotation data [58] were included as external data to complement the de novo transcript discovery. TSS coordinates were refined based on CAGE-seq, while the extension of transcript ends was defined based on signal change point analysis from GRO-seq, or if the available annotated gene region matched the candidate transcript the longer transcript region between the annotated and discovered transcribed region was kept (genomic overlaps were analyzed using BEDtools [59], for details see Methods from [9]). Finally, the pre-miRNA annotations from GENCODE 2018 [60] and miRbase v.20 [61] were used to annotate the subset of primary transcripts that overlapped miRNA coordinates. The coordinates were then converted to mm10 using UCSC liftOver tool [62] to be compatible with the most recent genome version and are Listed in Additional file 2: Table S1E and F for mouse and human, respectively.

### Building a custom transcript annotation for scRNA-seq gene quantification workflows

Typical scRNA-seq quantification workflows, including the 10x Genomics Cell Ranger pipeline [63], allow users to build their own custom transcript annotation based on existing reference annotation data that describes gene, transcript, and exon information. To develop a custom reference suitable to quantify the transcriptional activity of miRNA genes, we defined miRNA genes as the merged region starting from the most distal TSS mapped to the miRNA and extending until the longest transcript end, using the GRO- and CAGE-seq-based annotation for previously defined human coordinates [9] and mouse coordinates described above. This region was included in the GTF file as a single exon (pri-miRNA) transcript. However, also alternative transcript and exon structures have been experimentally defined for miRNA genes based on knockout of the key processing enzyme Drosha [64]. Therefore, the annotation was extended by adding these candidate alternative transcript structures and at annotated coding gene regions exons were included from GENCODE 2018 and 2013 for mouse and human genomes respectively [60, 65] motivated by manual examination of splicing patterns captured in 10x Genomics and Smart-seq2 scRNA-seq data. Cell Ranger requires that GTF files are preformatted using “cellranger mkgtf” command and that a FASTA file (reference genome) containing the nucleotide sequences of the selected transcripts is provided [63, 66]. The generated GTF with miRNA genes and the FASTA file for mouse mm10 or human hg19 genome were used as input for the “cellranger mkref” command. The additional quantification for the miRNA genes was combined in downstream analyses with the default GENCODE-based count matrix.

### TMS and Tabula Sapiens data

The TMS and Tabula Sapiens consortia generated single-cell libraries that were produced either using single-cell suspensions combined with droplet detection (10x Genomics) or by fluorescence-activated cell sorting (FACS) sorting individual cells combined with Smart-seq2 technology hereafter referred to as “Plate-seq” as denoted in the original TMS publications [12–14]. Selected files including Liver, heart and aorta, fat, and bone marrow tissues at 1 month, 3 months, 18 months, 21 months, 24 months, and 30 months of age were downloaded from the TMS Amazon cloud as described in the GitHub repository available for this work [67]. A List of samples including number of samples per tissue used can be found in Additional file 2: Table S1B and C. In this work, we focused on data from the spleen. To compare with human data, we retrieved matching human data from the Tabula Sapiens [48] and quantified read counts from miRNA gene coordinates.

10x Genomics datasets were downloaded in FASTQ format and processed with the default Cell Ranger pipeline (v.3.0.2) [63]*.* The detection of cell-containing droplets in 10x Genomics data was performed using the default count matrix, and the miRNA gene counts were added based on matching cell barcodes and quantified with the option –include-introns (v.6.1.1). Smart-seq2 [68] samples denoted as “Plate-seq” were downloaded in bam format and quantified with FeatureCounts (Subread package v.2.0.1, [69]) using the custom GTF reference genome with default options (in this case, the libraries are strandless). Droplets assigned by the Cell Ranger pipeline (v.3.0.2) as cell-containing droplets (filtered matrix) were further quality controlled, filtered by QC metrics and processed using a standard Single-Cell Analysis in Python (SCANPY)-based workflow described in [47, 70, 71] with minor modifications.

The average gene expression and miRNA detection rates were compared between the platforms using Spearman correlation calculated from B cells, T cells, and NK cells of splenic cells.

### Slide-tag data analysis

Currently, the droplet-based technology can also be performed in spatial context using the Slide-tag protocol [53]. To verify that miRNA gene expression can be analyzed from these profiles, we retrieved a published study (FASTQ files from hippocampus sample available in GSE244355 [54]), designated as spatial scRNA-seq, and quantified cell type-specific miRNA profiles using the custom miRNA reference with the 10x Genomics Cell Ranger pipeline (v.6.1.1). Subsequently, the data was combined with processed gene quantification results, meta-data, and spatial coordinates available from Broad Institute Single Cell Portal (SCP2170) [72]. The R Seurat package v.4.0.1 [73] was used to normalize the data. Marker genes for radial glial cells were identified based on the Wilcoxon test (FindMarkers), reporting both up- and downregulated genes relative to other cell types.

### Animal model of disease progression

LDLR^−/−^ApoB^100/100^ transgenic mice have a phenotype characterized by high accumulation of fat in the tissues as they lack the ability to remove circulating lipid particles using the low-density lipoprotein receptor (LDLR) [74, 75]. This model is commonly used to follow atherosclerotic plaque formation in veins and arteries, reflecting disease progression in humans. Full details on the atherosclerotic plaque development in this disease model including molecular characteristics are described in [76]. Here, we used male mice and an experiment setup where samples were collected at various disease progression stages. To capture the early disease (ED) state, the transgenic mice were fed a combination of a chow diet and after, a fat diet (HFD; 0.2% total cholesterol, Teklad TD.88137) for 1 month. To study the impact of elevated pro-inflammatory signaling on immune cell tissue infiltration during disease progression, mice following ED diet were injected with LPS during the HFD phase 2 weeks prior to sacrifice. Late disease (LD) state was achieved by feeding a fat diet for 3 months [77]. The transgenic mice fed with a chow diet represented the pre-lesioned state (PL) and C57BL/6 J mice on chow diet were used as an additional control in annotation of cell types. We timed the diet-starting age to equalize the age at sample collection between all groups (8 months old). Throughout the study, mice were maintained on a 12-h light–dark cycle and had access to food and water ad libitum.

### TRAP-seq animal model and capture of ribosomal transcripts

To study disease-associated translatomes in myeloid cells, we crossed the LDLR^−/−^ApoB^100/100^ with mice expressing cfms (Csf1r) promoter 3 × FLAG-EGFP-L10a transgenic construct (*n* = 6 per condition). The mice followed the same diet schedule as described above. Upon sacrifice, their tissues were frozen and pulverized using a cryopress, followed by lysis. Lysis buffer (1 ml per 100 mg of tissue) was freshly prepared, containing a low-salt/homogenization buffer (20 mM HEPES pH 7.4; Fisher, 10,041,703), 150 mM KCl (Sigma, 60,142), 10 mM MgCl_2_ (Invitrogen, AM9530G), supplemented with 0.5 mM dithiothreitol (DTT) (Sigma, 10,197,777,001), cOmplete Mini EDTA-free protease inhibitor (Roche 11,836,170,001, one mini tablet/10 ml), 100 μg/ml cycloheximide (Sigma-Aldrich, 1810) in dimethylsulfoxide (DMSO) (Sigma C1988-1G), 0.2 U/μl murine RNase inhibitor (NEB M0314L), and 0.1 U/μl Superasin RNase inhibitor (Thermo Scientific AM2696). The homogenized samples were centrifuged at 2000 × g for 10 min at 4 °C and the supernatant was collected, mixed with 10% IGEPAL CA630 (Sigma, I8896)] to reach final 1%v/v and with 1,2-diheptanoyl-sn-glycero-3-phosphocholine (Avanti Polar Lipids, 850306P) in final concentration of 30 mM. Samples were incubated on ice for 5 min centrifuged at 20,000 × g for 10 min at 4 °C.

Biotinylated Protein L (0.5 mg; Pierce #29,997) was dissolved in phosphate-buffered saline (PBS) and 120 µl mixed with 300 µl of Dynabeads MyOne Streptavidin T1 (10 mg/ml; Invitrogen 65,602) for pulldown experiments. The bead-protein complex was used to capture anti-GFP antibodies (clone 19F7 and clone 19C8, from the Antibody and Bioresource Core Facility at Memorial Sloan Kettering Cancer Center). Lysis buffer was freshly prepared, containing a low-salt/homogenization buffer (20 mM HEPES pH 7.4; Fisher, 10,041,703), 150 mM KCl (Sigma, 60,142), 10 mM MgCl2 (Invitrogen, AM9530G), supplemented with 0.5 mM DTT (Sigma, 10,197,777,001), cOmplete Mini EDTA-free protease inhibitor (Roche 11,836,170,001, one mini tablet/10 ml), 100 μg/ml cycloheximide (Sigma-Aldrich, 1810) in dimethylsulfoxide DMSO (Sigma C1988-1G), 0.2 U/μl murine RNase inhibitor (NEB M0314L), and 0.1 U/μl Superasin RNase inhibitor (Thermo Scientific AM2696). The buffer was added to the tissue at 1 ml per 100 mg of tissue, and homogenized samples were centrifuged at 2000 g for 10 min at 4 °C to separate debris. Supernatant was collected, mixed with 10% IGEPAL CA630 (Sigma, I8896) to reach final 1%v/v and with 1,2-diheptanoyl-sn-glycero-3-phosphocholine (Avanti Polar Lipids, 850306P) in final concentration of 30 mM. Samples were incubated on ice for 5 min centrifuged at 20,000 × g for 10 min at 4 °C.

### Tissue harvest and cell dissociation for scRNA-seq with hashtag antibody staining

To Minimize technical sample processing differences, we generated scRNA-seq profiles from harvested tissue pools, where initially samples from each mouse were incubated with a specific hashtag antibody to later verify sample origin. Mice were anesthetized with isoflurane and euthanized by cervical dislocation. The cardiac puncture was carried out with 10 ml of ice-cold PBS supplemented with 20 U/ml heparin and the mice were placed into ice for dissection to collect aorta, white adipose tissue (WAT) and spleen tissues. The cell isolation for scRNA-seq was carried out immediately, while remaining tissue was snap-frozen for bulk RNA isolation.

Approximately 200 mg of epididymal WAT of each mouse (*n* = 3 for each condition) was Minced and added to 2.5 µl of Miltenyi Adipose Tissue Dissociation solution (supplemented with bovine serum albumin (BSA) and HEPES) and incubated for 45 min at 37 °C on an end-over-end rotator. During dissociation, the tissue was triturated 3 times (at 25 min, 35 min, and 45 min) to break up cell aggregates. Tissue samples were passed through a 30-µm cell strainer and washed with 3 ml of RPMI. Cells were centrifuged at 400 g for 8 min at 4 °C and the pellet was resuspended in 300 µl of FACS buffer (PBS with 1% BSA). Red blood cells were lysed using 1X RBC Lysis Buffer, Multi-species (eBioscience #00–4300-54) by mixing 300 µl of cell suspension with 2.7 ml of ice-cold 1X RBC lysis buffer and incubating for 3 min on ice. Two milliliters of FACS buffer was added to normalize the buffer and the cells were collected by centrifugation at 400 × g for 8 min at 4 °C. After RBS lysis, the cells collected from each mouse were stained with TotalSeq Mouse Hashtag antibody-DNA conjugates (BioLegend) containing a unique barcode sequence according to the manufacturer’s recommendations. The cell pellets were resuspended with TotalSeq Mouse Hashtag Ab-DNA conjugate in FACS buffer. The cell suspension was incubated in ice for 15–20 min to allow for hashtag Ab binding. Afterwards, cells were washed two times with FACS buffer to remove unbound hashtag Abs. Dead cells were removed with Miltenyi Dead Cell Removal kit (Miltenyi Biotec #130–090-101) as described before [78]. The cell pellets were resuspended in PBS containing 0.04% BSA and counted by hemocytometry with trypan blue staining. Cell viability was between 74 and 85%. For each condition, approximately 18,000 cells (pooled from 3 mice) were loaded into the Chromium Controller microfluidics chip (10x Genomics).

Blood samples for monocyte isolation were processed by first performing erythrocyte lysis by mixing aliquots of 500 µl of EDTA blood with 4.5 ml of ice-cold 1 × RBC lysis buffer and incubating on ice for 3 min. Subsequently, cells were centrifuged at 500 × g for 5 min at 4 °C, supernatants were discarded, and erythrocyte lysis was repeated. The cells were washed with 5 ml FACS buffer, followed by staining with TotalSeq Mouse Hashtag Ab-DNA conjugates in FACS buffer for 15–20 min on ice. Following staining, the cells were washed once with FACS buffer and once with MACS buffer (PBS with 0.5% BSA and 2 mM EDTA). After staining, the CD115 + monocytes were enriched using Miltenyi Biotec MicroBead kit (# 130–096-354) as described by the manufacturer. The stained cells from individual mice (three per condition) were pooled in 90 µl MACS buffer, combined with 10 µl of the FcR Blocking Reagent (Miltenyi Biotec), and incubated for 10 min at 4 °C. Ten microliters of CD115-Biotin conjugates was added and the suspensions were mixed and incubated for 10 min at 4 °C and the cells were pelleted by centrifugation at 300 × g for 5–10 min at 4 °C. The supernatants were discarded, and the cell pellets were resuspended in 80 µl of MACS buffer. Twenty microliters of Anti-Biotin MicroBeads was added to the solution, mixed and incubated for 15 min at 4 °C. The cells were washed with 1–2 ml of MACS buffer and centrifuged at 300 × g for 5–10 min at 4 °C. The cells were resuspended in 500 µl of buffer. MS columns were placed in the magnet and the samples were passed through 30-µm cell strainers before applying them to the MS columns. All the subsequent Steps were performed at 4 °C. The columns were washed three times with 500 µl of MACS buffer. After removing the column from the magnet, 1 ml of elution buffer was added, and the cells were flushed out by firmly pushing the plunger into the column.

The cells collected from each tissue (including aorta not used in this study) were centrifuged at 300 g for 5–10 min at 4 °C, resuspended in PBS containing 0.04% BSA, and counted by hemocytometry with trypan blue staining. Cell viability was between 74 and 85%. For each condition, approximately 30,000 cells (pooled from 3 mice) were loaded into the Chromium Controller microfluidics chip (10x Genomics). ScRNA-seq Libraries were generated with the Chromium Single Cell 3′ v.2 assay (10x Genomics). Libraries were sequenced using the NovaSeq 6000 platform (Illumina) to a depth of approximately 300 million reads per Library with read lengths of 26 (read 1) + 8 (i7 index) + 0 (i5 index) + 91 (read 2).

### ScRNA-seq data integration and label transfer in the atherosclerotic mouse model

We used a standard data processing approach where raw reads were aligned to the mouse genome (mm10) using Cell Ranger (count pipeline) (v.3.0.2, [63]). Data was normalized using SCTransform [79, 80] to account for differences in sequencing depth in the Seurat R package v.4.0.1 for integration [73, 81–84]. We formed the reference dataset by the unbiased integration of the control (C57BL/6 J on chow diet) and late disease conditions for each tissue. The rest of the conditions were then added using the canonical correlation (CCA) algorithm (k.anchor = 20, dims = 1:50) [83]. Cell type predictions at the broad lineage level were obtained by performing tissue-wide label transfer [85]. WAT labels were transferred from the TMS fat dataset. Blood tissue labels were transferred from the 10x Genomics Peripheral blood mononuclear cell (PBMC) human reference dataset [63]. The LD vs. ED comparison was selected for myeloid cell subtype and statistical analysis to ensure sufficient cell number was available from both groups compared (nCells PL: 212, ED: 380, and LD: 1303). Gene symbols were translated to mouse with the biomaRt R package v.2.54.1 [78, 86].

### Data demultiplexing by hashtag signals and pre-processing in the atherosclerotic mouse model

The atherosclerotic mouse model samples were hashtag-barcoded with individual barcodes added to distinguish between mice. Tissues including WAT and aorta yielded initially very low signal in cells detected by Cell Ranger, resulting in ~ 80% negative cells with default demultiplexing settings (mostly in cells that were annotated as stromal cells). To overcome this limitation, we used the DSB R library v.1.0.3 [87] which was built to estimate the difference between the actual antibody signal in cell-containing droplets and the background signal in empty droplets in cellular indexing of transcriptomes and epitopes (CITE)-seq single-cell libraries. The DSB workflow uses the raw matrices containing all the droplets available and produces a matrix of scaled protein signals vs. the background signal [88]. We used hashtag scaled matrix as input to perform the hashtag-based demultiplexing. We also noticed that the differences in the distribution of hashtag signal intensity between cells of distinct lineage (e.g., myeloid vs. lymphoid) were causing errors in the annotation of doublets across the tissue (proportions of negative cells or doublets would not typically be expected to vary by cell type). To overcome this, we performed the hashtag demultiplexing separately by the broad cell lineage annotation obtained using label transfer. We continued the analysis with cells that were annotated as individual cells (singlets) and repeated the sample integration. To check the quality of the libraries generated, we followed a basic QC and filtering workflow using the SCANPY v.1.8.2 package for each tissue [89]. Transcripts were filtered to include those that were present in more than 3 cells. To assess the viability of the cells, we quantified mitochondrial and ribosomal genes. Cells were filtered out according to a maximum mitochondrial gene expression percentage, a maximum number of counts, and a minimum number of expressed genes. Due to heterogeneity in the raw data, these parameters were set for each tissue and condition (refer to Additional file 2: Table S1B). Expression data was normalized with size factor values derived from data normalization using the scran R package v.1.26.2 [90], and then log-transformed using the function scanpy.pp.log1p. Highly variable genes were calculated with scanpy.pp.highly_variable_genes, selecting the top 4000 genes for principal component analysis and dimensional reduction. Louvain and Leiden clustering at different resolutions were performed to define similar transcriptome states that can be used for assigning lineage and cell type annotations.

### Differential expression analysis using scDD

To compare miRNA expression levels across sample groups, we applied a variance stabilizing transformation (vst) normalization as a pre-processing step. This method, introduced by Hafemeister and Satija, borrows information across genes that have similar expression levels to improve the gene-specific estimates [79]. The strategy was evaluated through benchmarking experiments using TMS spleen data. First, to simulate a scenario with no true differential expression, we artificially generated two groups from male B cells aged 1–3 months (from a total of 2880 cells) (i) by randomly assigning 300 cells to each group, or (ii) by assigning 300 cells from lowest third of cells based on nCount and comparing them to 300 cells from the highest third nCount. The results with and without vst normalization were compared, with statistically significant differences identified using either the Wilcoxon rank sum test implemented in the Seurat R package v.4.0.1 package FindMarkers function or using the scDD package functions (see below). Secondly, to assess sensitivity of the test, we compared sub-sampling results with varying number of cells (300, 500, 1000, and 1500) to the result obtained using the full dataset.

Based on the specificity and sensitivity testing, the log2 counts based on vst transformation available in Seurat R package v.4.0.1 were used to compare differences in gene expression distributions in a given cell type between different sample groups (e.g., young and old, or late vs. early disease) using the scDD v.1.14 method [91, 92]. We carried out statistical analysis to detect miRNA gene expression changes based on the fraction of cells expressing a certain transcript (DZ category) and among expressing cells by comparing the expression level (DE, DP, and DM categories) following the approach described in [93, 94]. Each analysis included cells collected from multiple individual mice; details of these animals are provided in Additional file 2: Table S1B. Transcripts with adj. *p* value < 0.05 (Benjamini–Hochberg FDR method [95]) were considered significant. Combined *p* values were calculated based on Fisher’s exact test (sumlog function from metap R package v.1.8) across all cell types to identify miRNAs that were concordantly regulated during aging. From those, miRNAs with associated *p* values < 0.05 that passed a log fold change cut-off in at least two cell types were considered top-ranking candidates presented in the figures.

### Metacell analysis of tissue myeloid cell subpopulations

We generated an unbiased map of subpopulations for myeloid cells based on a similarity graph calculated using cells from white adipose tissue ED, IC, and LD samples (*n* = 329, 819, and 1098, respectively). Homogenous groups of cells denoted as “metacells” were identified with the MetaCell R package v.0.3.7 [96, 97]. Specifically, gene-level statistics were computed with “mcell_add_gene_stat” and featured genes were selected based on their variance (> 0.8, “mcell_gsetfilter_varmean”) and number of UMIs (> 100 UMIs in the entire dataset and selected genes are required to be detected in at least 3 cells with > 2 UMIs, “mcell_gset_filter_cov”). A balanced cell graph or “balanced K-nn graph” was computed as previously described (K = 100, “mcell_add_cgraph_from_mat_bknn”). Next, we performed resampling (*n* = 500) and generated the co-clustering graph (“mcell_coclust_from_graph_resamp,” min. node size = 20, cell partitions = 5.000 covering 75% of the cells). Metanodes assignment-derived statistics from the co-clustering step are evaluated with “mcell_mc_from_coclust_balanced” command with default settings. To check that the metacell nodes are homogeneous, cells that highly deviate from their metacell’s expression profile were plotted as outlier cells (“mcell_plot_outlier_heatmap,” data not shown) and filtered afterward “mcell_mc_split_filt.” Gene markers per node were extracted for further analysis with “mcell_gset_from_mc_markers” and metacells projected into a 2D graph for visualization (“mcell_mc2d_force_knn,” “mcell_mc2d_plot”).

### Differential expression analysis of bulk GRO-seq profiles from macrophageex vivo cultures

To verify transcriptional activation of miRNA gene loci, we used samples Listed in Additional file 2: Table S1D for differential expression testing. The data was processed as previously described in [9]. Low-expressed transcripts were filtered and differences in transcription level between sample groups were analyzed based on linear model fitting with limma and edgeR R packages [98, 99]. Changes in primary transcription levels at miRNA gene loci were assigned as significant based on adjusted *p* value < 0.05 (Benjamini–Hochberg method).

### TRAP-seq data analysis

We carried out a differential ribosomal-associated analysis of mRNAs comparing mice fed with a HFD for 3 months (LD, *n* = 6) vs. mice fed with a chow diet (PL, *n* = 6). The TRAP-seq samples were pre-processed as described in [100]. First, the data distributions were compared and samples with the lowest sequencing depth (< 7 million reads) were excluded from the analysis. Subsequent linear model fitting was carried out as described for the GRO-seq datasets. Genes were ranked based on *p* values and the subset of genes passing a nominal *p* value cut-off of 0.05 was submitted to the Mienturnet platform [101] to perform miRNA target gene enrichment analysis based on miRNA-target interactions from the TargetScan database [102]. MiRNA target enrichments were assigned as significant based on an adjusted *p* value < 0.1 (Benjamini–Hochberg method).

### Mature miRNA quantification by RT-qPCR

To study changes in mature miRNA expression of selected miRNA, total RNA from flash-frozen WAT and spleen samples (WAT (all conditions *n* = 6 except LD *n* = 3), spleen (*n* = 6 per group), and blood monocytes (data from pool of 3 animals)) was isolated using the TRIsure reagent (Bioline), following the manufacturer’s protocol with Minor modifications. TRIsure was added directly onto the tissue: 800 µl for samples weighing 30–50 mg and 1 ml for samples > 50 mg. Samples were homogenized in TRIsure with a bead using TissueLyser II (Qiagen, 30 Hz, 45 s, 2 cycles). Following homogenization, samples were centrifuged at 12,000 × g for 10 min at 4 °C. The aqueous phase was transferred to a new tube and chloroform was added in proportion to the volume of TRIsure used, followed by phase separation. RNA was precipitated by adding ice-cold isopropanol, and samples incubated overnight at − 20 °C. The next day, RNA was pelleted by centrifugation at 14,000 × g for 30 min at 4 °C. To isolate RNA from flash-frozen 150,000 monocytes, 500 µl of TRIsure was added directly to the cell pellet, without homogenization or overnight precipitation. UltraPure™ Glycogen (Invitrogen) was added at a concentration of 10 µg per sample to facilitate RNA precipitation. All WAT and monocyte samples were spiked with external RNA controls (Qiagen, Cat.no. 339390) at a ratio of 1 µl per 1 ml of TRIsure. Reverse transcription (miRCURY LNA RT Kit, Cat.no. 339340) and quantitative PCR (miRCURY LNA SYBR Green PCR Kit, Cat.no. 339345 and 339,346) were performed using the Qiagen miRCURY® LNA® miRNA SYBR® Green PCR system, following the manufacturer’s protocol (Qiagen, miRCURY LNA miRNA SYBR® Green PCR Handbook, version 10/2019). A custom RT-qPCR panel (Qiagen, GeneGlobe ID YCA46081, Cat.no. 339330 and 339332) was used to detect the spike-in controls and mature target miRNAs. Reactions were run for 45 cycles on a Roche LightCycler® 480 instrument. Data analysis was performed using the Qiagen GeneGlobe analysis platform. Raw Ct values were first corrected using the plate calibration control UniSP3, followed by correction with internal amplification controls (UniSp6, cel-miR-39-3p, and UniSp2). Ct cut-off 35 was used as a lower limit of detection. Expression levels were normalized using three reference miRNAs (hsa-miR-103a-3p, hsa-let-7d-5p, and RNU5G), which showed consistent expression of miRNAs and snoRNAs across different disease states. For statistical testing, an unpaired two-tailed *t*-test was selected within the GeneGlobe analysis platform. A fold-regulation threshold of 1 and a *p* value threshold of 0.05 were applied. Relative expression of mature miRNAs was calculated using the 2^(− Avg(ΔCt)) method and visualized in R version 4.1.0 using the ggplot2 package (version 3.3.5).

## Results

### Quantification of miRNA gene expression in single-cell transcriptomes

**Fig. 1:**
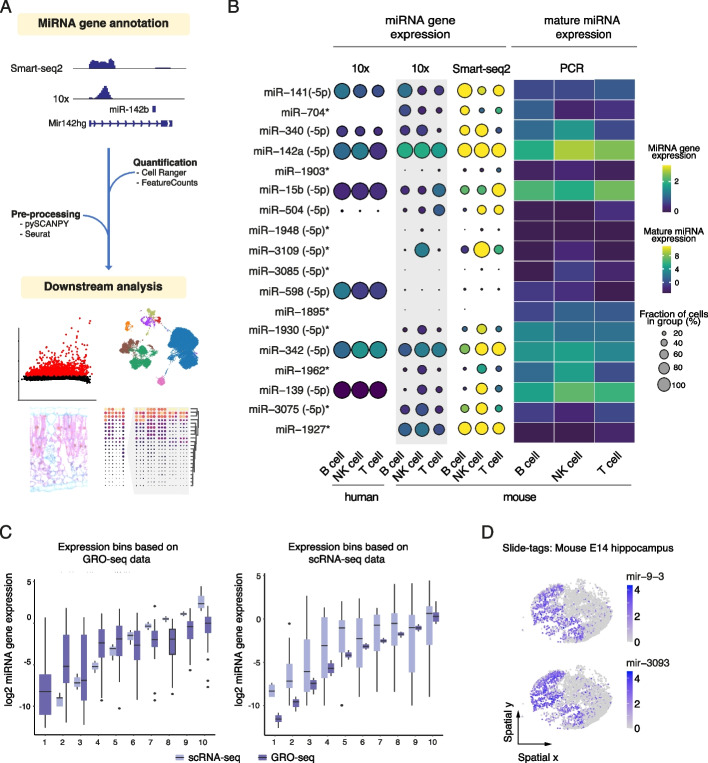
MiRNA gene annotation and quantification in scRNA-seq datasets. **A** Overview of the miRNA gene quantification for scRNA-seq data. Gene and miRNA gene counts were extracted with the Cell Ranger or FeatureCounts pipelines, followed by downstream analysis performed by combining Seurat [73] and SCANPY [71] packages to obtain and study cell type-specific miRNA gene expression at single-cell resolution. **B** MiRNA gene markers (*n* = 6 per cell type, defined from mouse 10x data in gray shade) were compared to their corresponding human genes and mature forms in splenic cells including B cell, NK cell, and T cell subpopulations. On the left, dot plot heatmap shows miRNA gene expression (from high expression in yellow to low expression in blue) and percentage of expression (circle size) based on droplet-based (10x Genomics) and FACS-coupled Smart-seq2 technology. On the right, the heatmap depicts mature miRNA expression levels from FACS-sorted splenic cells measured with PCR (GSE144081 from [50]). * mouse miRNA genes that were not annotated in the human genome. **C** Comparison of scRNA-seq-based quantification (log2 miRNA gene expression) to GRO-seq-based primary transcript expression in the mouse stromal cell line ST2. Genes were binned from low to high expression levels based on GRO-seq data (lower panel) or scRNA-seq data (upper panel). **D** Spatial miRNA expression analysis in mouse hippocampus. Significant marker genes for radial glia are shown

To characterize miRNA gene expression dynamics in single-cell transcriptomes collected from mouse tissues, we first followed the approach described in [9] to obtain mouse transcript coordinates representing intergenic miRNA genes transcribed from their own promoter and intragenic miRNA co-transcribed from introns of their host genes (see Methods, datasets used are Listed in Additional file 2: Table S1A). For simplicity, transcripts from alternative transcription start site (TSS) were summarized by miRNA gene locus, following the current practice of capturing gene-level expression in single-cell datasets (see Methods, similarly human coordinates were adopted from [9]). The resulting annotation of 233 intragenic and 135 intergenic miRNA gene loci (corresponding to 990 mature miRNAs for mice) and 511 and 391 loci, respectively, for humans (1896 mature miRNA), was integrated into the GENCODE 2018 transcript annotations (Fig. 1A, Additional file 2: Table S1E and F: miRNA gene coordinates).

Next, we retrieved a comprehensive dataset on aging mice from TMS consortium [12–14], which we used as a benchmark for miRNA gene quantification at cellular resolution across tissues. The TMS single-cell transcriptomes were sequenced with 10x Genomics and Switching Mechanism at the 5′ end of RNA Template (Smart)-seq2 technologies (Additional file 2: Table S1B and C). While both methods follow a poly-A-based priming strategy, 10x Genomics uses 3′-based quantification whereas Smart-seq2 captures reads along the entire transcript. To determine which method more accurately captures miRNA gene expression dynamics, we quantified read counts from miRNA gene coordinates in splenic cells from 3-month-old mice from both platforms (matching human data was retrieved from Tabula Sapiens [48]). We further studied miRNA gene dynamics based on the ability of each platform (i) to measure miRNA gene expression levels and (ii) the ability to detect miRNA quantified as “percentage of expression.”

The two platforms had comparable performance in detecting different miRNA genes: across cell types, on average 59 miRNA were found with 10x Genomics 3′RNA-seq (hereafter 10x Genomics) and 66 with Smart-seq2 (Additional file 1: Fig. S1A). Overall, expression profiles across the three main splenic cell types (B cells, T cells, and NK cells) correlated well between the two platforms when the average expression level (Spearman rho at cell type level 0.60–0.72) or the detection percentage (Spearman rho at cell type level 0.88–0.91) were compared (Additional file 1: Fig. S1B).

To study the relationship between miRNA genes and their corresponding mature forms in individual transcriptomes, we identified miRNA gene markers (*n* = 6) for the main splenic immune cell populations including mouse B, T, and NK cells using the statistical tests for cluster comparison in the SCANPY pipeline (Fig. 1B, dot plot panels). We then retrieved their corresponding mature forms and quantified their expression from polymerase chain reaction (PCR)-based profiles available from the immune cell atlas (GSE144081 from [50]; Fig. 1B, heatmap right panel). Relative expression levels among the platforms, across species, and between the gene and corresponding mature forms were concordant for highly expressed genes, exemplified by (mmu)-miR-141 with highest expression in B cells and mmu-miR-340 with high expression in NK cells and B cells in relation to T cells (Fig. 1B, notice that miRNA loci marked * did not have corresponding human data). Overall, the correlations between the percent of expression or average expression in single-cell analysis and mature miRNA expression were generally weaker (0.44–0.47 in all the comparisons). Since the mature miRNA levels could be affected by transcript processing and stability, we performed a complementary and more direct benchmark comparison to primary transcription assayed using bulk GRO-seq and parallel 10x Genomics scRNA-seq in the mouse stromal cell line ST2 (see Methods, Fig. 1C). MiRNA genes were divided into 10 bins based on their expression in each sequencing technology (plotted from low to high values in Fig. 1C, Light purple indicates 10x Genomics-based scRNA-seq and dark purple GRO-seq signal). Independently of the data type used to bin miRNA genes, the detected expression level was highly comparable at bins of high expression (bins from 6 to 10), whereas the bins corresponding to lower expression levels displayed higher variability. This observation agrees with Limitations in efficiently capturing low-expressed transcripts in 10x Genomics scRNA-seq datasets [7]. Taken together, quantifying miRNA gene expression based on droplet and plate-based scRNA-seq chemistries has high concordance with cell-specific bulk expression data. Since more cell types were detected from 10x Genomics-based profiles, we continued with this technology in downstream analyses to capture miRNA transcription at cellular resolution.

As a final benchmark, we confirmed that our approach is compatible with the 10x Genomics-based spatial platform Slide-tags [53], exemplified by radial glia cell-specific miRNA marker genes identified from mouse E14 hippocampus [54] (Additional file 2: Table S1G). The most significant markers included mmu-mir-9 that has a well-established developmental role [103] and a novel candidate regulator mmu-mir-3093 (Fig. 1D, Additional file 1: Fig. S1C).

### Aging profiles in splenic immune cells reveal coordinated and cell type-specific changes in miRNA gene expression

**Fig. 2:**
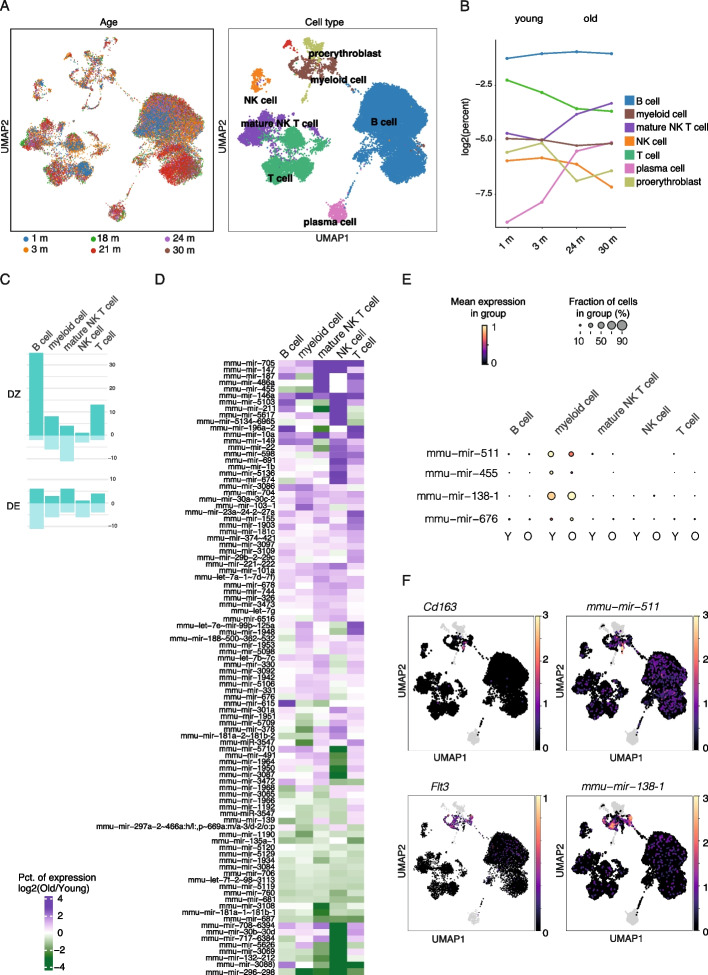
Differential expression analysis of miRNA genes in spleen tissue during aging. **A** Uniform manifold approximation and projection (UMAP) plot of the integrated spleen (all age groups, left) dataset (*n* = 27,260 cells). Colors indicating different cell types from 8 major clusters (*n* > 150 cells) (annotations from TMS) include B cells (*n* = 18,398) in dark blue, T cells (*n* = 4029) in forest green, mature NK T cells (*n* = 2131) in purple, myeloid cells (macrophages, *n* = 652 and dendritic cells, *n* = 439) in brown, NK cells (*n* = 385) in orange, proerythroblast (*n* = 464) in light green, plasma cells (*n* = 559) in pink, and dendritic cells (*n* = 249) in red*.* **B** Cell type percentages from male mice present in each age group of the dataset are shown as a line plot. **C** The number of DZ and DE miRNAs detected in each cell type separated by up and downregulated genes are shown (dark green and light green, respectively). **D** Heatmap showing top-ranked miRNA genes that were differentially detected (DZ) across main cell types in the spleen (Fisher’s exact test associated *p* value < 0.05, absolute log2 FC > 0.5). Fold changes between old and young cells are shown in each cell type. **E** Dot plot heatmaps of myeloid-specific changes in miRNA gene levels (adjusted *p* value < 0.05). **F** UMAP plots of macrophage and dendritic cell gene markers (on the left) and cell type-specific miRNAs (on the right)

During aging, the immune system deteriorates, manifesting in loss of homeostatic mechanisms controlling immune responses that can underlie chronic inflammation and thereby risk for developing various aging-related diseases. To gain insights into miRNA expression profiles in aging immune cell subpopulations, we retrieved mouse samples from the TMS consortium, the largest resource of single-cell datasets to study aging in multiple tissues. We focused on the splenic male samples that covered the broadest range of time points. The annotation contained seven major splenic cell types that cluster together independently of age (Fig. 2A and Additional file 1: Fig. S2A). During aging, mature NK T cell and plasma cell proportions increased whereas myeloid cells, proerythroblast, T cell, and NK cell proportions decreased (Fig. 2B and Additional file 1: Fig. S2C), in line with previous results [12].

To identify a robust strategy for detecting Changes in miRNA gene expression, we benchmarked different normalization and statistical testing strategies with TMS 10x Genomics data from B cells based on different test scenarios (outlined in Methods). Both scaling by total counts and vst transformation were robust against false positives in the random cell selection (young vs. young): no significant miRNA expression changes (adjusted *p* value < 0.1) were reported by the Wilcoxon ranked sum test, or the scDD tests that distinguished each cell type changes in the expression level within cells expressing the transcript (DE category) and variations in the percentage of cells expressing a particular gene (DZ category). When selection was biased by nCounts, we observed a similar FP percentage with Wilcoxon ranked sum test with (300 cells: 6%; 500 cells: 13%) and without (300 cells: 5%; 500 cells: 10%) normalization. The vst normalization reduced FP for scDD (300 cells: from 18 to 3%; 500 cells: from 22 to 4%, where FP were almost exclusively from DE category). Accordingly, we chose to apply vst normalization to improve specificity of downstream statistical analyses.

Next, we compared within each cell type young (1 and 3 months) and old mice (24 and 30 months) corresponding to sufficient time difference [14] to detect progressive, gradual Changes in gene expression. In total, 131 DZ and 58 DE miRNA genes were identified across the different cell types, summarized in Fig. 2C and Additional file 3: Table S2. For a sensitivity benchmark, we sub-sampled different cell numbers to both groups and compared the results of each test using all data. The TP percentage for Wilcoxon ranked sum test increased from 12% with 300 cells per group to 50% with 1500 cells, while recovery of TP scDD DE increased from 43 to 84% and scDD DZ from 2 to 52% (Additional file 1: Fig. S2A). Based on the favorable specificity and sensitivity performance, we selected scDD for downstream analyses. The comparison of the average expression and detection rates of significant vs. non-significant miRNA genes (Additional file 1: Fig. S2B) confirmed that changes in miRNA levels were captured across a broad expression range and not driven by low-expressed genes. Next, miRNA genes were ranked based on combined *p* values (Fisher’s exact test, see Methods) to identify concordant Changes among the five most abundant immune cell types, which revealed 187 and 42 significant (combined *p* value < 0.05) miRNAs in DZ and DE categories, respectively. A subset of 94 top-ranked miRNA with log2 fold change in detection rate > 0.5 in two cell types are shown in Fig. 2D (refer to Additional file 1: Fig. S2C–E for corresponding cell proportion and miRNA expression profiles in female samples).

The miR-29 and 30 family microRNAs that promote senescence (inhibition of DNA synthesis by targeting B-myb [104]) and mir-155 were previously recognized as global aging markers in TMS small RNA-seq bulk analysis [105]. In agreement, we found upregulation of mmu-mir-30a ~ 30c-2 and mmu-mir-29b ~ 29c in several cell types and the loci ranked high based on significance in the Fisher test. We also found several previously aging-associated miRNAs, including mmu-mir-146a and mmu-mir-147 that were concordantly upregulated in the immune cell types and upregulation of mmu-mir-101a in lymphoid cells (T cells, B cells, and NK T cells) of old mice (Fig. 2D, top), in agreement with their established role in aging brain tissue [106], and regulating NF-κB and Toll-like receptor (TLR) mediated inflammatory responses senescence [107, 108], respectively. Notably, their mature forms did not reach significance in the previous bulk small RNA-seq analysis. Upon closer examination, female samples comprising young (3 months) and adult mice (18 and 21 months) only showed common upregulation of mmu-mir-147 and a decrease of mmu-mir-146a and mmu-mir-101a (Additional file 1: Fig. S2D), likely underlying the discrepancy. Further expression changes seen in aged immune cells that may aggravate aging phenotypes include the downregulation of mmu-mir-706 (Fig. 2D, bottom) with recognized function as an oxidative stress regulator [109] and the concordant increase in mmu-mir-705 (regulation of aging-related cell fate bias [110]).

Unique to our analysis, we could identify highly cell type-specific changes, exemplified by myeloid cell-specific changes (Fig. 2C, E and Additional file 1: Fig. S2E). The observed decrease in mmu-mir-455 transcription may perturb its aging-protective function in limiting hypoxia-inducible factor-2α expression [111]. Similarly, the fraction of cells expressing mmu-mir-511 involved in the regulation of TLR-signaling decreased. In contrast, we observed upregulation of mmu-mir-138–1 and mir-676 transcription towards aging in myeloid cells. Previously a similar pattern towards aging has been reported in keratinocytes where miR-138 promotes cellular senescence via targeting *Sirt1 *[112]. These myeloid-lineage-specific changes matched specific subpopulations of cells, corresponding to dendritic cells and macrophages (refer to Fig. 2F showing the respective marker genes: *Flt3* for antigen-presenting dendritic cells and *Cd163* for macrophages).

Taken together, our analysis revealed aging-related transcriptional changes of miRNA genes involved in regulatory networks governing senescence, oxidative stress, and inflammatory responses, distinguishing several miRNAs impacted in multiple immune cell types and providing the resolution to detect highly cell type-specific expression.

### Myeloid cell compartment expansion marks disease development in fat tissue upon high-fat diet

**Fig. 3:**
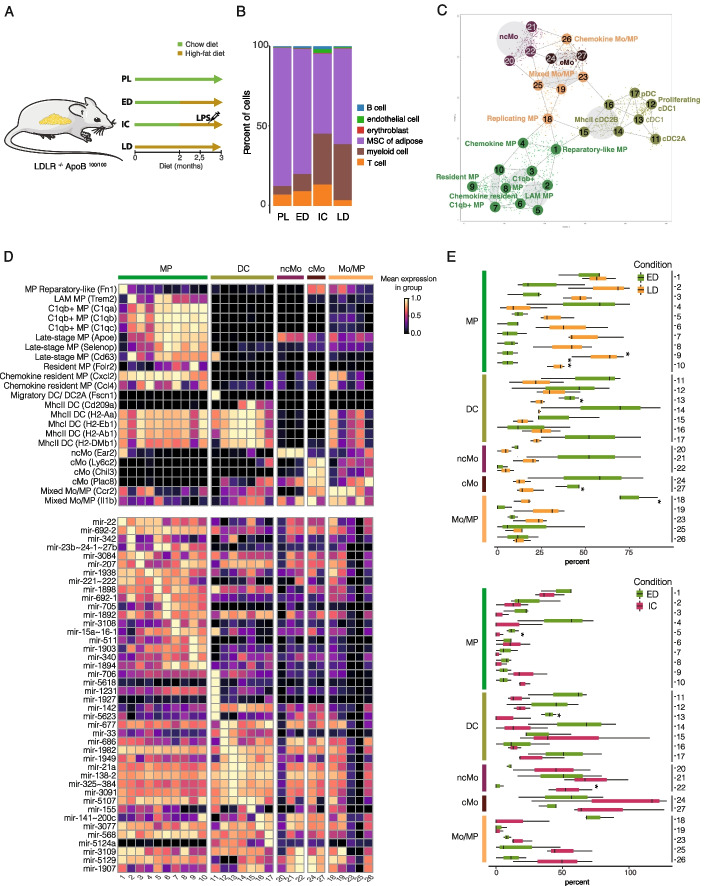
Adipose tissue myeloid cell subpopulations and their miRNA gene profiles during atherosclerosis disease progression. **A** Scheme of the study atherosclerosis mouse model. Mice were fed either a chow diet (in green) or fat diet (in gold). Samples were denoted as early disease (ED), inflammatory challenge (IC), and late disease (LD) for LDL deficient mice. **B** Proportion of cell types across the sample types. **C** Cells grouped in meta nodes based on their reproducible phenotypes. **D** Heatmap showing expression of selected node marker genes (above) and marker miRNA genes (below). Expression is row-scaled. **E** Changes in cell proportions between ED and LD conditions (above) and between ED and IC conditions (below)

An unhealthy diet is a risk factor for disease development that can result in elevated white adipose tissue (WAT) inflammation through altered cytokine and chemokine secretion in which specific immune cells are key players [113]. To model this process and analyze changes in miRNA gene expression, we collected scRNA-seq profiles from conditions representing progressive atherosclerosis development in male LDLR^−/−^ApoB^100/100^ mice fed with a chow (PL, pre-lesioned) or high-fat diet (*n* = 3 per group, QC data shown in Additional file 1: Fig. S3A). The experimental setup led to atherosclerotic plaque formation phenotype [76, 77] resembling early disease state (ED, shorter fat diet) and late disease (LD, longer fat diet resulting in advanced vascular lesions) (Fig. 3A), confirmed by examining the vessel wall cell phenotypes at lesions [76, 77]. In addition, we included lipopolysaccharide (LPS) as an extra inflammatory stimulus introduced during the fat diet (2 weeks prior to tissue collection in ED condition) to achieve an inflammatory challenged state (IC) (Fig. 3A). In response to IC and at LD, the proportions of immune cells (T cells, B cells, and myeloid cells shown in Fig. 3B) were modulated, with concomitant decrease in relative proportion of non-immune tissue-resident stromal cells.

The myeloid cell fraction increased the most between different conditions (Fig. 3B). To provide an in-depth analysis of the myeloid compartment upon disease progression, we defined reproducible cell phenotype states by computing a cell similarity graph from the ED, IC, and LD scRNA-seq profiles using the MetaCell pipeline. Next, we annotated the 27 nodes and found that each represents a unique transcriptome state (Fig. 3C), using marker gene detection and literature-based markers (Fig. 3D, Additional file 1: Fig. S3B, Additional file 4: Table S3A).

Macrophage (MP) markers were highly expressed in nodes 1–10. Specifically, node 1 had elevated expression of genes such as *Retnla* and *Fn1* (termed the reparatory-like MPs). Node 2 showed elevated expression of *Trem2* which is a well-known marker of lipid-associated macrophages (termed the LAMs) [114]. Nodes 3 and 5–10 showed a higher expression of genes encoding for innate immune protein *C1q* expression that in macrophages was previously suggested to alleviate inflammation present during atherosclerosis disease progression [115]. Accordingly, cells matched to nodes 5–10 predominantly represented the LD condition and showed a relatively higher expression of late-stage MP markers (*Apoe*, *Selenop*, and *Cd63* [116]). In addition to node 2, nodes 5 and 6 had elevated expression of *Trem2*, indicating a LAM-like transcriptional signature. Node 9 showed a high expression of *Cd163*, *Lyve1*, and *Folr2* (termed as the tissue-resident MPs [117]). Interestingly, nodes 4 and 7 were enriched in genes associated with chemokine-signaling such as *Cxcl2* and *Ccl4*, suggesting that node 4 is composed of chemokine MPs and node 7 is composed of *C1q* + MPs with also chemokine secretion. Additionally, node 7 showed an intermediate expression of *Cd163* and *Folr2* further implying a tissue-resident-like MP phenotype.

Node 11 showed a distinct transcriptional state with a high expression of *Ccr7*, *Ccl22*, and *Fscn1* (Fig. 3D, Additional file 1: Fig. S3B). Cell populations enriched with these markers have previously been termed the migratory dendritic cells (DC) [116] and the classical DC2A [117]. *Xcr1* and *Clec9a* were highly expressed only in nodes 12 and 13 suggesting cDC1 phenotype [116, 118]. Additionally, node 12 was enriched in genes encoding the members of Cdc45/Mcm2-7/GINS (CMG) complex (*Mcm5* and *Mcm6*); thus, this node was termed as proliferating cDC1. Nodes 14, 15, and 16 had elevated expression of *Cd209a* together with major histocompatibility complex (MHC) II class genes (*H2-Eb1*, *H2-Ab1*, *H2-Aa*, *H2-DMb1*) and therefore defined as MHCII DC [116]. Plasmacytoid DC markers (*Siglech*, *Ccr9*, *Cox6a2*, *Atp1b1*, *Ly6d* [116]) were exclusively expressed in node 17.

Node 18 showed a mixture of different signatures: monocyte-derived MP (*Ccr2*), interferon (INF) (*Isg15*), chemokine (*Cxcl10*), and active DNA replication (*Top2a*) suggesting that they are actively replicating MPs undergoing transition potentially towards INF or chemokine MPs (Fig. 3D). Markers of mixed Mo/MP (*Ccr2* together with *Fcgr1*, and *Itgam* [116]) were present in node 19. Since nodes 20–22 showed upregulation of *Ace* and *Ear2* together with downregulation of *Ccr2* and *Ly6c2*, they were defined as non-classical monocytes (ncMo [116]) (Additional file 1: Fig. S3B). In contrast, nodes 24 and 27 showed upregulation of *Ccr2* and *Ly6c2* together with high expression of *Chil3* and *Plac8*, and these nodes were thus defined as classical monocytes (cMo [116]). Nodes 23 and 25 showed mixed patterns of Mo and MP such as intermediate expression of *Ccr2* (Mo-derived MP), *Isg15*, *Isg20* (INF), *Fcgr1*, and *Itgam* (Early MP) (Fig. 3D). Node 26 was enriched in genes associated with INF signatures such as *Isg15* and *Isg20* and was therefore defined as mixed Mo (Additional file 1: Fig. S3B).

### miRNA gene markers for myeloid subpopulations and disease progression

Comparison of relative cell proportions revealed that macrophage nodes 1–3, 5–10, 19, and 23 had a high representation of cells in LD condition whereas ncMo nodes 20–22 and cMo/Mo-MP nodes 24–27 were predominant in the IC condition (Fig. 3E, Additional file 1: Fig. S3C). We next examined miRNA gene expression specific to the metacell subpopulations. Highly node-specific expression of several immunomodulatory miRNA genes distinguished the macrophage subtypes, in comparison to more subtle subtype-level differences between DC and monocyte (Mo) cell subtypes (Fig. 3E). The macrophage miRNA markers included the highest mmu-mir-22 levels (regulation of pro-inflammatory cytokine expression [119]) within reparatory MP, LAM-specific expression of mmu-mir-23b ~ 24–1 ~ 27b (downregulated miRNA in patients with autoimmune diseases that regulate pro-inflammatory cytokine expression [120]), high mmu-mir-221 ~ 222 in *Cq1* + MP and mmu-mir-15a ~ 16–1 (regulating phagocytosis [121]) in MP node 7. The most distinct miRNA gene signature among DC was found in cDC2 (including mmu-mir-706 with possible unconventional nuclear function [122]) and in n15 cells corresponding to MHCII DC phenotype, which express mmu-mir-155, known to function as a “master regulator miRNA” in DCs and MPs [123]. In DCs, mmu-mir-142a has a key role in regulating pro-inflammatory cytokines [124]. In agreement, our analysis identified it as a broadly expressed DC marker miRNA.

Next, we compared the miRNA gene expression distributions in ED and LD conditions in myeloid cells.

**Fig. 4:**
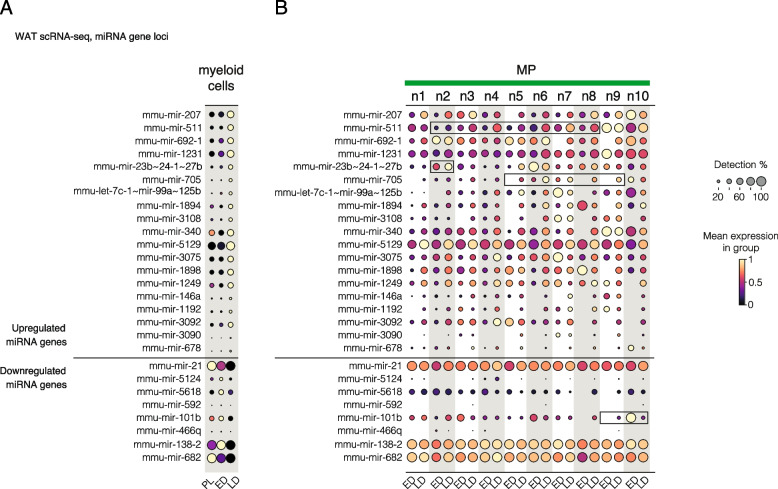
Disease progression alters miRNA gene expression in macrophage subpopulations. miRNA genes with altered expression during disease progression (**A**) and stratified by metanode (**B**) are shown as dot plot heatmap. Brighter color tone and larger dot size denote higher expression

Across all cells, our analysis identified 19 upregulated and 8 downregulated miRNA genes (Fig. 4, Additional file 5: Table S4) in both pro- and anti-inflammatory regulatory pathways with distinct expression in across disease stage (Fig. 4A, levels in PL shown for comparison) and across macrophage subtypes (Fig. 4B, see also Additional file 1: Fig. S4 for monocyte and dendritic cell nodes). Among immunomodulatory miRNAs with the potential to aggravate tissue inflammation, we noted increased expression of mmu-mir-511 [125] driven by nodes 2–8 (Fig. 4B). An opposite change was observed for mmu-mir-101b expression (a negative regulator of pro-inflammatory response [126]), with strongest repression in nodes 23 and 25 (nodes panel, Fig. 4B). In comparison, immunosuppressive mmu-mir-23b ~ 24–1 ~ 27b expression increased in Trem2 and Trem2-like MPs (nodes 2 and 5) (nodes panel, Fig. 4B) and miRNA genes encoding classical nuclear factor kappa B (NF-κB)-modulating miRNAs mmu-mir-146a and mmu-mir-21 [145] increased in expression in several MP nodes, mmu-mir-146a most strongly in node 7.

### Changes in miRNA gene expression produce a disease-associated translatome signature

**Fig. 5:**
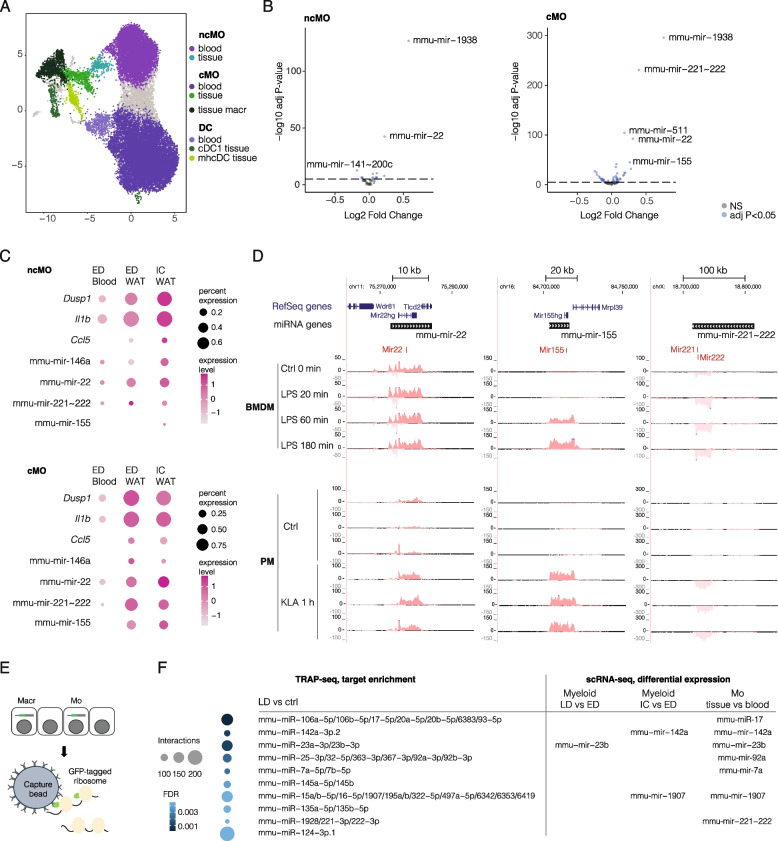
Gene expression changes in monocytes infiltrating adipose tissue relative to naïve blood precursors. **A** UMAP of blood and tissue (WAT) myeloid cell populations. **B** Volcano plots representing significant miRNA gene loci from tissue vs. blood comparison of monocyte subtypes. Top miRNA loci are indicated on the plot. FC corresponds to differences in detection rate. **C** Dot plot heatmap comparing known LPS-responsive genes to the profile of top miRNA loci in tissue and blood. **D** GRO-seq signal tracks at mmu-mir-22, mmu-mir-155, and mmu-mir-221 ~ 222 loci. The + strand signal is shown above the vertical axis (dark red tone) and − strand signal below (light red tone). NcMo: non-classical monocyte, cMo: classical monocyte, DC: dendritic cell, ED: early disease, IC: inflammatory challenge, BMDM: bone marrow-derived macrophage, PM: peripheral macrophage, LPS: lipopolysaccharide, KLA: Kdo2-lipid A. **E** Schematic illustration of TRAP-seq with myeloid-specific capture of translated mRNAs. **F** Ten most significantly enriched miRNA seed sequences in TRAP-seq are compared to observed miRNA gene expression changes detected from scRNA-seq. Blue color tone indicates FDR (darker is more significant) and dot size the number of DE gene associations.

The prominent increase of myeloid cells in adipose tissue at late disease, or at early disease upon LPS stimulus, prompted us next to examine changes in gene regulation during myeloid maturation into tissue-resident cells. We hypothesized that the tissue cytokine environment could trigger changes in gene regulation and thereby miRNA expression. Thus, we collected additional scRNA-seq profiles from blood monocytes (Additional file 1: Fig. S5A) and integrated these with the myeloid cell profiles from WAT. The monocytes in the blood are typically short-lived, representing a reference naïve state for the comparison. The cells obtained from blood and WAT clustered primarily based on their tissue-of-origin (Fig. 5A, UMAP), however with similar subpopulations (ncMO; cMO; DC) from both tissues placed adjacent to each other, as defined using marker genes (Additional file 1: Fig. S5B, see also Fig. 3 and Additional file 1: Fig. S3). We focused on the main monocyte and DC subtypes and performed a statistical comparison of their tissue vs. blood expression profiles (Fig. 5B, Additional file 6: Table S5A and B, refer also to Additional file 1: Fig. S5C for DC comparison). In total, our analysis detected significant Changes in 45 and 95 miRNA genes in ncMo and cMo, respectively. Among the top upregulated miRNA genes, mmu-mir-1938 and mmu-mir-22 are highly upregulated in both monocyte types, while the most significant changes in cMo (more pro-inflammatory monocyte type) include also upregulation of mmu-mir-221 ~ 222, mmu-mir-511, and mmu-mir-155. The comparison of top miRNAs and classical LPS-responsive genes (*Dusp1*, *Il1b*, *Ccl5*) in blood and WAT (Fig. 5C) highlights that the miRNAs associate with a pro-inflammatory transcriptional signature (dot size and darker red color tone) that is further elevated in IC condition, most prominently in ncMo.

To validate that the changes detected from scRNA-seq profiles represent regulation of the transcriptional activity at pri-miRNA loci, we used GRO-seq profiles (see Methods) collected from two different experimental setups: ex vivo LPS stimulation of bone marrow-derived CD14 + macrophages (referred to as BMDM) and LPS stimulation of peritoneal MPs (referred to as PM, resembling tissue-resident MP). Three intergenic miRNA gene loci highlighted in the scRNA-seq analysis (Fig. 5A and B) are shown in Fig. 5D. The elevated GRO-seq signal levels within the gene regions confirm that upregulation of mmu-mir-22 and mmu-mir-221 ~ 222 transcription occurs rapidly in both BMDM and PM cultures and remains high 180 min after LPS stimulus (see Table S5C and D for differential expression statistics and summary). In comparison, mmu-mir-155 is significantly upregulated with a delay at 60 min in BMDM, representing a more immature cell model. In peritoneal cells, its gene regulatory dynamics were comparable to the two other miRNA loci, overall, in agreement with their increased expression in the tissue microenvironment and following LPS stimulation in the in vivo scRNA-seq profiles.

Finally, to characterize how the modulation of miRNA expression may impact target gene mRNA translation, a key mechanism through which mature miRNAs regulate gene expression, we performed the TRAP-seq assay with pulldown from WAT Csf1r-expressing myeloid cells (monocytes and macrophages), comparing chow to a 3-month high-fat diet (Fig. 5E). To associate the respective expression Changes with miRNAs, we performed miRNA target enrichment analysis, resulting in 47 significant (FDR < 0.1) associations (Additional file 6: Table S5E). Notably, seven out of 10 miRNAs with the most significant target gene enrichment in the translatome are encoded by genes found regulated in the monocyte single-cell analysis (Fig. 5F). The disease-associated increase in LAM reflects as target gene enrichment signature for mmu-miR-23b and mmu-miR-27b (mmu-miR-24–1 nominally significant) that match the same pri-miRNA locus, while both tissue monocytes and LAM expression could contribute to mmu-miR-221 ~ 222 target enrichment (Figs. 3 and 4). In validation, we carried out measurement of mature miRNA levels at bulk tissue level in WAT, spleen, and isolated blood monocytes using RT-qPCR analysis from matched animals, selecting miR-17-5p and miR92a-3p encoded by mmu-mir-17; miR-142a-3p, miR-23b-3p, and miR-27b-3p encoded by mmu-mir-23b; and miR-7a-5p, miR-1907, and the mature forms miR-221-3p and mmu-miR-222-3p encoded by mmu-mir-221 corresponding to the most significantly enriched miR seeds. We also included mmu-miR-155 with lower significance (but passing FDR < 0.1) and mmu-miR-24-3p (FDR = 0.13) encoded by the same primary transcript with mmu-miR-23b and 27b but did not pass the FDR cut-off, and the alternative 5p forms mmu-miR-23b-5p and mmu-miR-221-5p to control strand-specificity. Expression of miRNAs selected for the validation analysis in WAT and blood scRNA-seq data by cell type and sample group is shown in Additional file 1: Fig. S6A and B. The mature miR profiles highlight the high and disease state-modulated expression of miR-142a and mmu-miR-23b-3p (with similar trend for −27b and −24) in WAT (no change in spleen) (Additional file 1: Fig. S6C). The early upregulation of miR-142a was also visible in the blood monocyte profiles in both ED and IC conditions (Additional file 1: Fig. S6D), while upregulation in LD was specific to WAT. In addition, 4/7 miRNA with lower expression were significant in WAT, while the miR mature forms matching the TRAP enrichment (miR-23b-3p and miR-221-2p) were clearly distinguishable from their strand controls by expression level. Among them, miR-155 was significantly upregulated also in spleen (Additional file 1: Fig. S6E). Taken together, our results demonstrate that disease development strongly modulates cell type-specific miRNA gene expression and that several of the changes observed at the transcriptional level show a target enrichment signature in the disease translatome.

## Discussion

MiRNAs are key modulators in maintaining tissue homeostasis by post-transcriptionally regulating gene expression. Thus, an imbalance in the expression of several miRNAs has been associated with disease progression and aging-related cellular processes including senescence [127, 128]. Here, we aimed to study cell type-specific miRNA gene expression during aging and disease progression. First, we demonstrated the feasibility of capturing miRNA gene expression from common droplet- and plate-based platforms and provided primary transcript annotations for mouse and human genomes that can be added to scRNA-seq quantification pipelines. Based on this approach, we quantified and analyzed miRNA gene expression in the TMS dataset, providing a unique pre-computed resource of cell type-specific miRNA aging signatures. Finally, we carried out a comprehensive genome-wide analysis of in vivo scRNA-seq, translatomes, and ex vivo nascent transcription profiles to confirm myeloid-specific miRNA regulation during disease progression. The upregulation of miR-23b and miR-221/222 in tissue monocytes and Trem2 macrophages and the increased prevalence of these cell populations were identified as key changes impacting the disease-regulated translatome.

The results presented in this study provide the first comprehensive benchmark evaluating the most commonly used scRNA-seq platforms for the quantification of miRNA gene expression. Droplet-based approaches, represented here by the 10x Genomics technology, capture a larger number of cells, allowing a better estimation of the heterogeneity of a cell population [7, 129]. The Slide-tags method employing a similar droplet-based approach allows miRNA gene analysis even in the spatial context, as demonstrated here. When strand-specificity is lacking, Smart-seq2 analysis has similar limitations as chromatin immunoprecipitation (ChIP)-seq-based analysis of transcriptional activity, described in [10]. Plate-based studies, on the other hand, often aim at higher sequencing depth per cell and have full gene body signal coverage. This could benefit the characterization of differences in alternative transcripts within miRNA gene loci. The main limitation in both commonly used platforms is that the detection of miRNA gene transcription suffers from a limited number of intronic reads captured. For this reason, previous miRNA gene analysis have focused on scRNA-seq profiles generated from nuclei that provide higher capture of intronic reads [130] and thereby improve miRNA gene detection [131]. As we demonstrate, even the 3′ platforms provide insight into miRNA regulation, enabling wide adoption of the proposed approach. Nevertheless, the limitations posed by dropouts should be carefully considered in the normalization and statistical analysis steps, to reliably detect changes in low abundance miRNAs. We opted to combine miRNA genes with regular genes in upstream steps where scaling factors are calculated and applied the variance stabilization algorithm proposed in [79] in the statistical analysis to avoid biases originating from expression level differences. Other choices to mitigate this issue could be tested in future, such as pooling counts across cells into pseudobulk analysis. To carry out the analysis, we assembled miRNA gene annotation defined using integrated GRO-, CAGE-, and ChIP-seq and Drosha knockout (KO) profiles for both human and mouse genomes. Alternative transcript assembly-based approaches [132] have been proposed, yet these leverage the read data only partially, and the feasibility is impacted by insufficient cell numbers per cell type. Quantification of miRNA genes based on the pre-defined coordinates as we propose here readily extends to other data types, including transposase-accessible chromatin (scATAC-seq) and scMultiome data analysis pipelines that facilitate the analysis of cell type-specific gene regulatory network activity orchestrated by transcription factors [133]. Our approach allows the use of miRNA gene expression as a proxy of mature miRNA expression in single cells, which is still technically challenging [6, 134, 135]. However, post-transcriptional events including primary miRNA processing and formation of an active RNA-induced silencing complex (RISC) influence the relationship between miRNA gene transcription and the presence or function of mature miRNAs (e.g., [136–141]). Despite these challenges, combining transcriptional data with analyses of post-transcriptional regulation and mature miRNA signatures offers a more comprehensive view of gene regulatory networks controlling tissue homeostasis.

Aging is a stepwise process characterized by changes in tissue homeostasis and cellular heterogeneity. In this study, we performed comparisons of miRNA gene expression in immune cells from different tissues, including bone marrow-derived blood cells and more mature splenic and tissue-resident populations. Our analysis identified altered expression of several miRNA genes (mmu-mir-101, mmu-mir-29/30) in the spleen that have an established function in the regulation of immunosenescence and apoptosis that throughout the body presents with alterations of immune cell homeostasis and an overall decline in immune efficacy [142]. The two TMS atlases (scRNA-seq cell-specific annotation generated here and bulk small RNA-seq profiles from [105]) afford the opportunity to extend these comparisons to additional tissues. We limited the statistical comparisons in scRNA-seq profiles to male mice, which is different from earlier comparisons that included both sexes. Our more conservative choice relates to the lack of representation of both sexes in certain age groups, with more time points available in male mice with sufficient time difference. For example, in the spleen tissue, our analysis of male data included young (1 and 3 months) versus old (21 and 30 months) comparison, while the female data corresponded to adult (18 and 21 months) versus young (3 months) comparison. Concordantly, the profiles from our atherosclerotic mouse model represent male mice, enabling comparison to TMS data without a confounding sex effect. However, the different aging profile of mmu-mir-146a in females suggests that miRNA gene regulation is impacted by sex and is in agreement with a recent human study reporting that the miR‐146a age-related trajectory was confirmed only in men [143]. Therefore, in future studies, it would be important to collect data representing more comprehensively both male and female biology. More broadly, sex biases in immune responses are well-established and known to strongly influence disease prevalence [144, 145].

Aging populations represent a global challenge and therefore new approaches to predict and prevent the progression of age-related functional changes are urgently needed [146]. Mature miRNAs are highly stable and therefore miRNA gene expression profiles could inform biomarker studies for monitoring disease progression. New therapeutic approaches based on miRNA-delivery into tissues are in development, with a promise to reduce the burden of aging and immune dysfunction-related diseases including type 2 diabetes, atherosclerosis, and dyslipidemia. Previous studies have highlighted changes in the adipose depot distribution along the body that dramatically affect tissue growth, plasticity, and function leading to metabolic dysfunction and low-grade inflammation [147]. The cross-talk between adipose tissue and immune cells is crucial for both the maintenance of normal healthy adipose tissue function and systemic metabolism [148]. Disease progression triggered by HFD led to the expansion of the myeloid compartment in WAT, identifying the miRNA expression in monocytes, macrophages, and dendritic cells as a potential target for future therapeutic intervention. Guided by unbiased clustering of cells into nodes, we demonstrated that WAT myeloid cells differ in miRNA gene expression, with the highest basal miR-511 levels in tissue-resident macrophages (nodes 9 and 10) and increased expression of this miRNA gene across multiple subtypes upon disease progression. Traditionally, macrophages were categorized as pro-inflammatory (M1) and anti-inflammatory (M2) macrophages, and high miR-511 expression was detected in M2 macrophages both in vitro and in vivo [149]. However, several studies have suggested this dichotomy to be obsolete and that M1 and M2 stages rather represent the extremes of a spectrum in a multidimensional space [150], reflected also in our results. Recent human studies have confirmed Trem2 + lipid-associated macrophages in obesity disease progression [117]. Here, we found that the highest expressed miRNA genes in Trem2 + macrophages were mmu-mir-221 ~ 222 and mmu-mir-23b ~ 24–1 ~ 27b loci. Their elevated expression at tissue level was further supported by the mature miR quantification. Previous functional studies demonstrated that miR-221/222 can inhibit adipogenesis and prevent diet-induced obesity [151]. Furthermore, the mmu-mir-23b ~ 24–1 ~ 27b locus encodes miR-23b, miR-27b, and miR-24–1 which each has a central role in the regulation of lipid metabolism [152–154] and glucose tolerance [155]. Thus, strong support from KO studies and the unbiased genomics profiling carried out in this study strongly implicate these miRNAs and the respective myeloid cell populations expressing them as disease-associated changes that may represent candidates for future interventional studies aiming to restore glucose and lipid metabolism balance.

In addition, infiltration of immune cells into tissues, especially those with pro-inflammatory function, is a known hallmark of both aging and disease development. miR-155 that we found upregulated in WAT and spleen at late disease induces pro-inflammatory activation of monocytes and through increased expression of human leukocyte antigen (HLA)-DR in the myeloid cells, modulates activation of T cells, and can thereby aggravate tissue inflammation, promoting disease progression in inflammatory-disease such as arthritis [156]. However, the induction of mmu-mir-155 coincided with elevated expression of miRNA genes typical of M2-like cells, such as miR-146a and miR-511. Together with miR-682, these miR converge in regulating NfKB activation. Our analysis associated their upregulation in myeloid cells with both aging and disease development, in line with earlier mouse studies that have shown that controlling NF-kB can modulate the onset of age-related symptoms and pathologies, as reviewed in [157]. In addition, we identified several less well-characterized miRNAs, including mmu-mir-1938, that merit future functional characterization. The top target prediction for miR-1838 is *Laptm5* (TargetScanMouse 7.1), encoding a lysosomal protein that modulates pro-inflammatory signaling in macrophages [158].

Presently, technologies are still immature to integrate cell-specific mature miRNA profiles. For this reason, we could not account for concomitant changes in RNA processing that occur during aging, including decreased levels of the miRNA processing enzyme Dicer both in mouse and human [159]. Once these approaches are available, their combination with TMS and similar aging atlases can expose the impact of miRNA dysregulation at further mechanistic detail. In this study, the analysis of miRNA expression upon disease progression was technically not feasible in aorta. The addition of other atherosclerosis-relevant organs and female animals would be important to include to future studies. The TRAP-seq assay used here for in-depth analysis of myeloid cells provides a complementary solution and a genome-wide approach to distinguish miRNA with an impact on cell type-specific translatomes. However, it requires a transgenic animal model or cell culture to introduce the cell-specific GFP-tagged ribosomes. Alternatively, integrating immunoprecipitation of argonaute family members followed by RNA sequencing or reporter-based screens [160] could serve as a strategy in future studies to reach a comprehensive understanding of how miRNAs collectively impact immune-cell interactions and tissue homeostasis.

## Conclusions

Our work represents the first unbiased and genome-wide evaluation of miRNA loci at cellular resolution in the aging mouse context. We focused on immune cell-specific changes in spleen and provided a data resource and new tools for single-cell genomics research to define miRNA regulatory networks with coordinated and cell type-specific activities across tissues. This study also examined in detail the adipose tissue disease trajectory, focusing on the transcriptional and translational regulation in myeloid cells that identified miR-23b and miR-221/222 and post-transcriptional regulatory network changes aligning with monocyte maturation as the key disease progression-triggered responses.

## Supplementary Information

Additional file 1: Supplementary Figures S1–S6 and supplementary table legends

Additional file 2: Table S1

Additional file 3: Table S2

Additional file 4: Table S3

Additional file 5: Table S4

Additional file 6: Table S5

## Data Availability

The new data generated in this study have been deposited to the NCBI Gene Expression Omnibus and Zenodo repositories with the following accession codes: GRO-seq data GSE241550, https://www.ncbi.nlm.nih.gov/geo/query/acc.cgi?acc=GSE241550, 10.5281/zenodo.16752187, https://zenodo.org/records/16752187, 10.5281/zenodo.16754687https://zenodo.org/records/16754687, 10x Genomics scRNA-seq data GSE241567, https://www.ncbi.nlm.nih.gov/geo/query/acc.cgi?acc=GSE241567, GSE241552 https://www.ncbi.nlm.nih.gov/geo/query/acc.cgi?acc=GSE241552, TRAP-seq data GSE254396 https://www.ncbi.nlm.nih.gov/geo/query/acc.cgi?acc=GSE241552. Refer to Data collection section and Additional file 2: Table S1, for sample identifiers and other public dataset accession codes. Data analysis codes for data pre-processing are available under GitHub repository https://anahsg.github.io/scMIR/ including links to.h5ad files comprising quantified scRNA-seq data objects with miRNA genes. We provide pre-analyzed miRNA gene expression profiles for >100000 cells from TMS (93217 cells) and Tabula Sapiens (17129 cells) to facilitate further characterization of miRNA regulatory networks in aging and across species as interactive interactive publicly available web resource at [161] https://kana.rahtiapp.fi/.
