# Supplementary material for: MicroRNA gene dynamics in immune cell subpopulations during aging and atherosclerosis disease development at single-cell resolution
Source: Genome Med. 2025 Oct 6;17:112. doi: 10.1186/s13073-025-01530-9 (PMC12502174; doi:10.1186/s13073-025-01530-9)
Supplement: Supplementary file 1 — Additional file 1: Supplementary Figures S1–S6 and supplementary table legends [file 13073_2025_1530_MOESM1_ESM.pdf]

Additional data file 1: Supplementary Figures S1-S6 and supplementary table legends.

miRNA gene dynamics in immune cell subpopulations during aging and atherosclerosis disease development at single-cell resolution

Ana Hernández de Sande[1], Tanja Turunen[1], Maria Bouvy-Liivrand[1,2], Tiit Örd[3], Senthil Palani[4], Mari Lahnalampi[1], Celia Tundidor-Centeno[1], Heidi Liljenbäck[4,5], Jenni Virta[4], Henri Niskanen[6], Buddika Jayasingha[1], Olli-Pekka Smålander[2,7], Lasse Sinkkonen[8], Lea Mikkola[9,10], Thomas Sauter[8], Anne Roivainen[4,5,9], Tapio Lönnberg[9,10], Minna U Kaikkonen[3] and Merja Heinäniemi[1]

[1] School of Medicine, University of Eastern Finland, Kuopio, North-Savo 70200, Finland
[2] Department of Chemistry and Biotechnology, Tallinn University of Technology, Tallinn 12616, Estonia
[3] A. I. Virtanen Institute, University of Eastern Finland, Kuopio, North-Savo 70200, Finland
[4] Turku PET Centre, University of Turku and Turku University Hospital, FI-20520 Turku, Finland
[5] Turku Center for Disease Modeling, University of Turku, FI-20520 Turku, Finland
[6] Department of Genome Regulation, Max Planck Institute for Molecular Genetics, Berlin, Germany
[7] Department of Neurology, Helsinki University Hospital, Helsinki, Finland
[8] Department of Life Science and Medicine (DLSM), University of Luxembourg, 4362 Belvaux, Luxembourg
[9] InFLAMES Research Flagship Center, University of Turku, FI-20520 Turku, Finland
[10] Turku Bioscience Centre, University of Turku and Åbo Akademi University, FI-20520 Turku, Finland

Table of contents

Supplementary table legends, Tables S1-S5

Supplementary figures S1-S6 and their legends

Supplementary table legends, Tables S1-S5

Additional file 2: Table S1. miRNA gene coordinates and related NGS dataset accession codes

(A) The datasets used for defining genomic intervals for miRNA gene custom quantification. (B-C) 'Dataset accession codes' for 10x and SMART-seq2 'scRNA data used in this study. NCBI GEO accession codes are listed. Cut-off parameters used in quality filtering of scRNA-seq data collected from $LDLR^{-/-}ApoB^{100/100}$ mice are also provided. (D) Additional validation genomics data accession codes. (E-F) 'miRNA gene coordinates' in mm10 and hg19 genomes. (G) Marker genes identified from mouse hippocampus. Related to Fig. 1.

Additional file 3: Table S2. Spleen scRNAseq summary of differential gene expression across immune cell types analyzed

Differential distribution analysis summary from comparison of old vs young male mice. The statistics for differential detection rate (DZ) (A) and expression level (DE) (C) of miRNA genes are provided separately for each cell type. Includes also ranking of miRNA based on Fisher test combined p-values across cell types (B and D for DZ and DE categories). Related to Fig. 2.

Additional file 4: Table S3. Atherosclerosis mouse model scRNA-seq data pre-processing settings and marker genes of WAT myeloid sub-populations

(A) Marker protein-coding and miRNA genes for metanodes defined from myeloid sub-populations in WAT are listed. Related to Fig. 3.

Additional file 5: Table S4. WAT scRNAseq summary of differential gene expression in myeloid cells at late disease

Differential distribution analysis summary from comparison of late disease vs early disease in $LDLR^{-/-}ApoB^{100/100}$ mice. The statistics for differential detection rate (DZ) (A) and expression level (DE) (B) are provided for myeloid cell comparison of miRNA genes. Related to Fig. 4.

Additional file 6: Table S5. WAT and blood myeloid cell scRNAseq and GRO-seq summary of differential gene expression

Differential distribution analysis summary from comparison of tissue vs blood monocyte and monocyte-derived cell populations in $LDLR^{-/-}ApoB^{100/100}$ mice. The statistics for differential detection rate (DZ)(A) and expression level (DE)(B) are provided for miRNA genes. (C) GRO-seq DE analysis of *ex vivo* cultured bone marrow- derived CD14+ or peritoneal macrophage stimulated with pro-inflammatory LPS or KLA treatments. (D) TRAP-seq miRNA seed enrichment analysis in myeloid cell translatome of $LDLR^{-/-}ApoB^{100/100}$ mice. Mierturnet result based on TargetScan. (E) Mature miRNA RT-qPCR summary. Related to Fig. 5. and Figs. S5-S6.

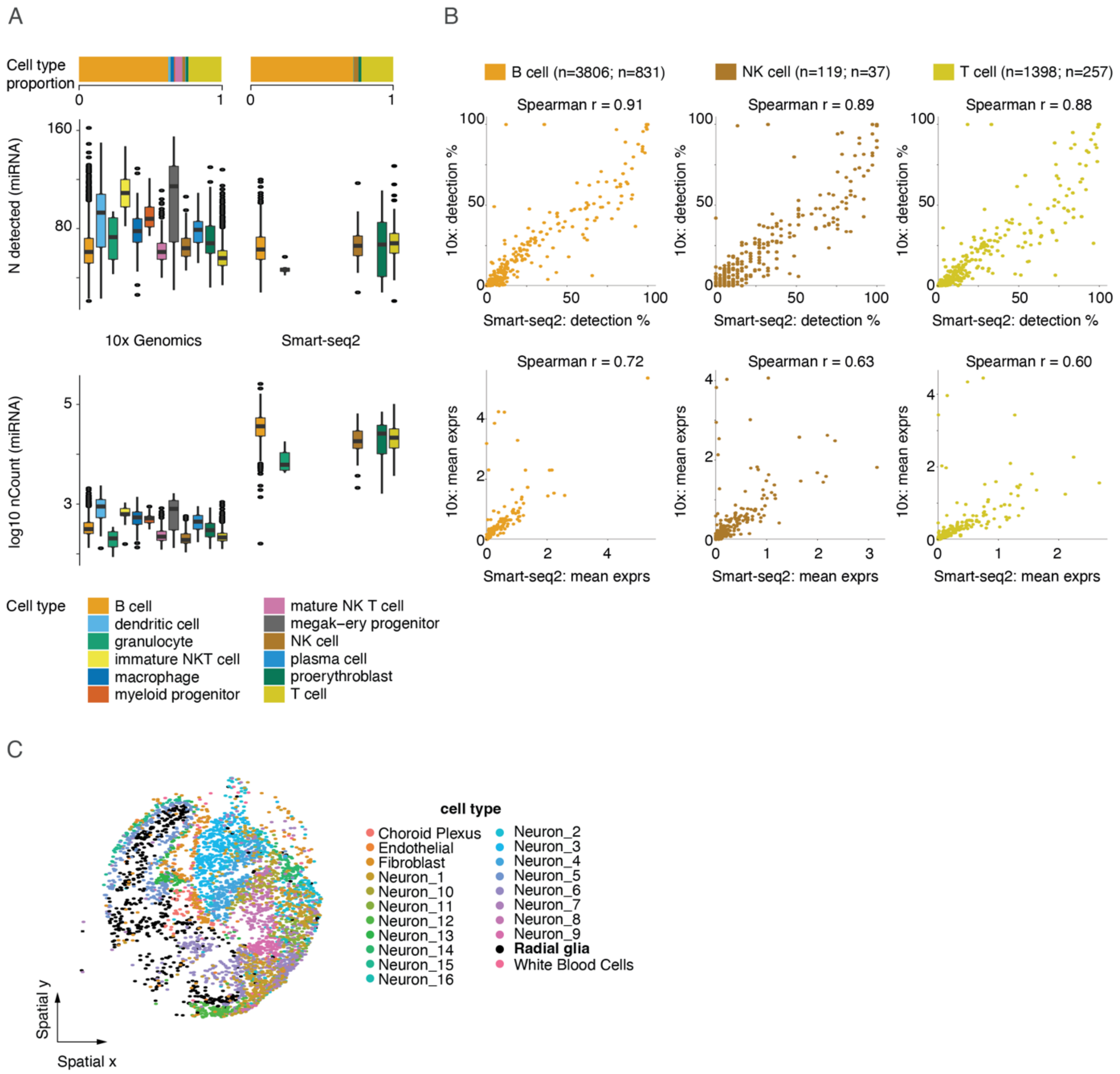

Fig. S1. Comparison of miRNA gene expression measured from 10x Genomics and Smart-seq2 scRNA-seq technologies using TMS spleen profiles.
(A) Boxplots showing the number of miRNA genes detected (upper panel) and their expression levels (lower panel) by cell type in 10x Genomics (left) and Smart-seq2 technologies (right). The relative cell type proportions are shown as barplot (top). (B) The correlation of 10x and Smart-seq2 miRNA levels based on detection percentage (above) and mean expression level (below) is shown for miRNA genes in three cell types. The cell numbers (nCells 10x; nCells Smart-seq2) and Spearman correlation coefficients are indicated above. (C) Cell type annotation for spatial transcriptomics data.

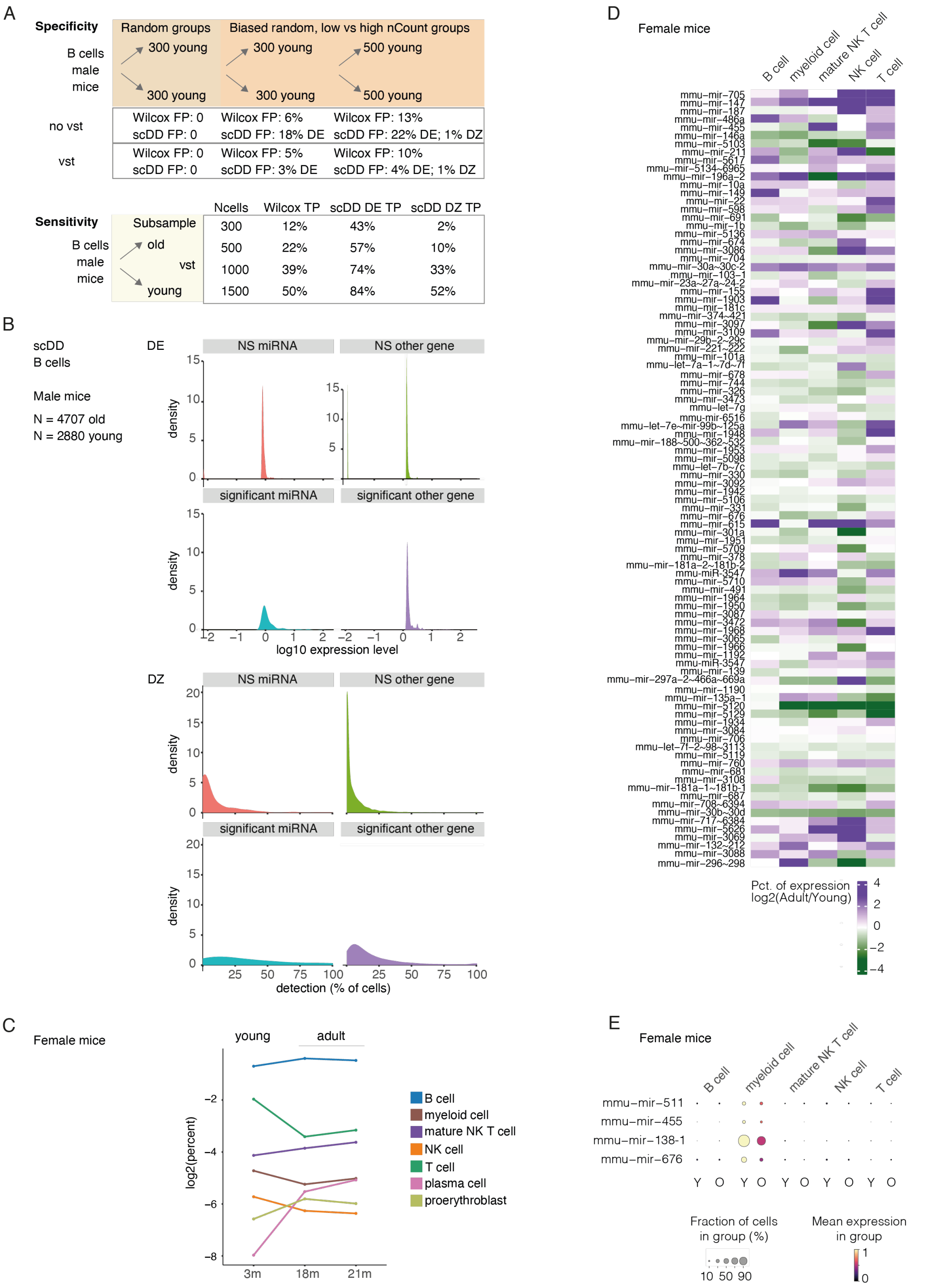

Fig. S2. Statistical analysis of splenic cell types in TMS data. (A) Specificity and sensitivity test results comparing different choices for normalization and statistical test performed. Results shown correspond to random simulations where B cells were randomly allocated to two groups either from young mice only (specificity test, upper panel, any significant genes are false positive, FP) or from old and young mice (sensitivity test, lower panel, genes matching result will all cells used were considered true positives, TP). vst: variance stabilization normalization; no vst: scaling total counts normalization. For the latter analysis, random sampling was repeated with n=300, n=500, n=1000 and n=1500 cells selected. (B) Density plots showing the log expression level (above) and detection percentage (below) in splenic B cells for comparison of miRNA genes (left) and other genes (right) stratified by statistical significance in the scDD analysis (male mice, number of cells in each age group is indicated on left and corresponds to the number of cells used for sub-sampling in experiments shown in panel A. (C) Cell type percentages from female mice present in each age group of the dataset are shown as a line plot. (D and E) miRNAs found concordantly regulated in splenic male samples during aging and plotted from female samples comprising young (3 months) and adult (18 and 21 months) mice as in Figs 2D and E.

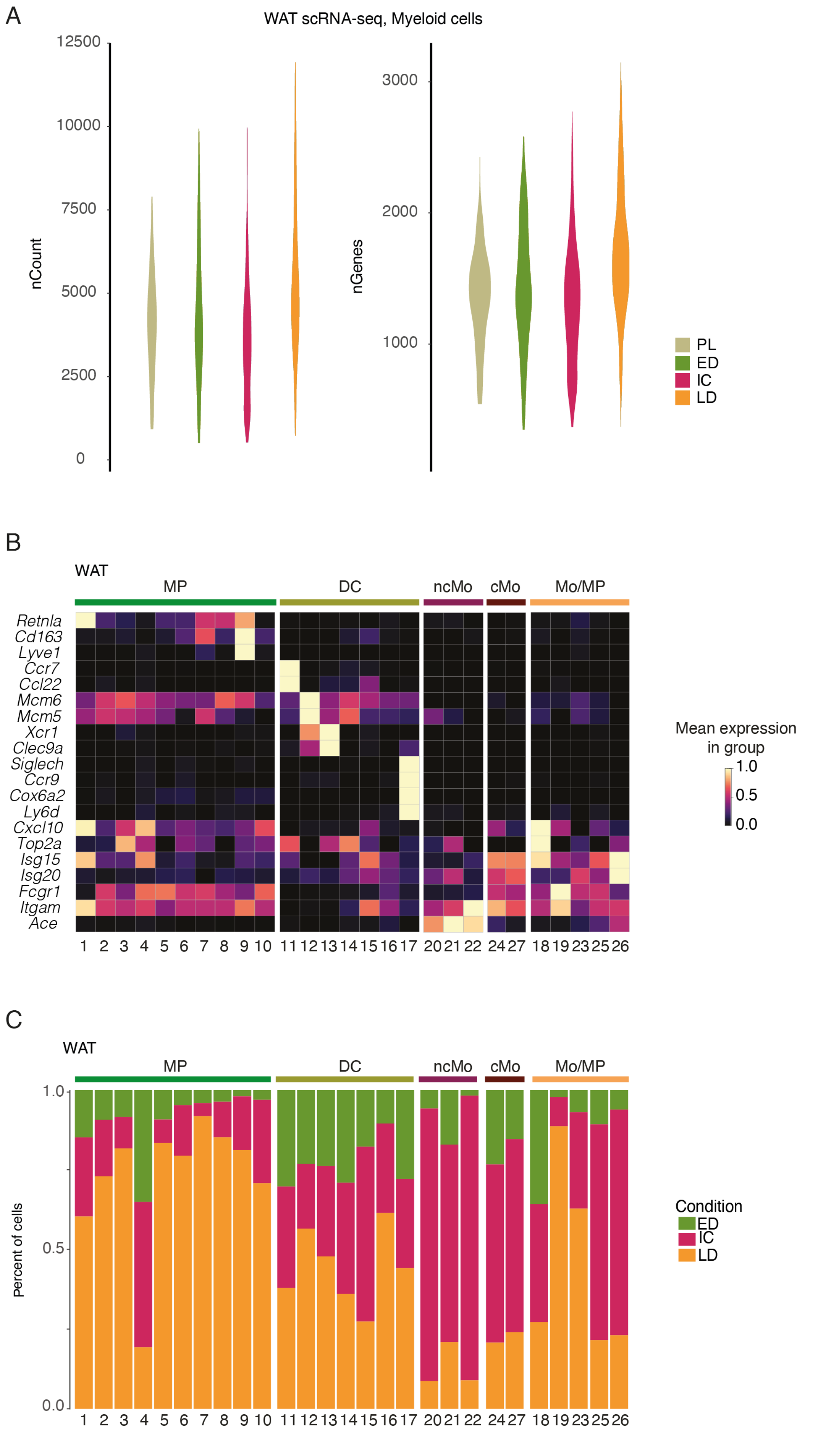

Fig. S3. Myeloid cell characterization in WAT during disease progression. (A) Quality control metrics (nCount: sequencing depth, nGenes: detected genes) for myeloid cells across sample groups shown as violin plots. (B) Heatmap showing expression of literature-based gene markers in nodes. Expression is row-scaled. (C) Proportions of cells representing different conditions in each node.

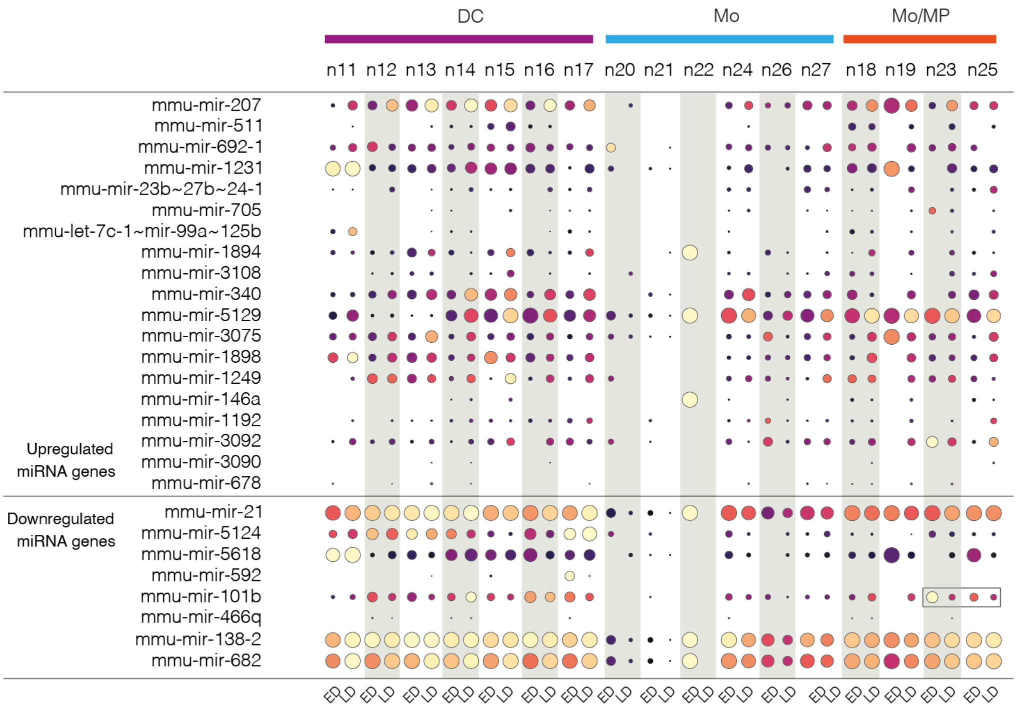

Fig. S4. Changes in miRNA gene expression during disease progression in WAT dendritic cells and monocytes. MiRNA genes with altered expression during disease progression are shown as in Fig. 4.

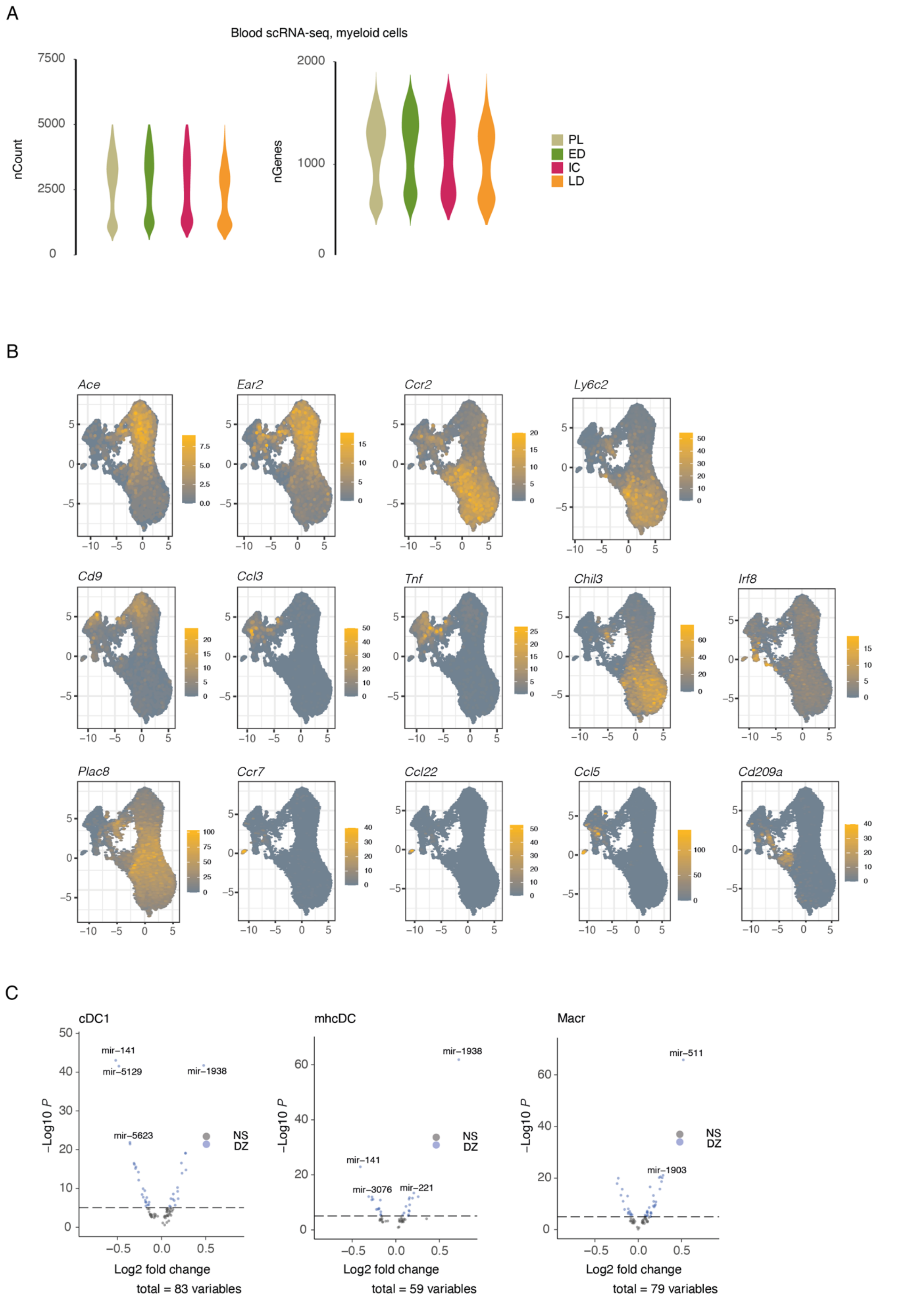

Fig. S5. Blood and tissue myeloid cell characterization. (A) Quality control metrics for myeloid cells across sample groups in blood tissue shown as violin plots. (B) Marker gene expression shown on UMAP representing the tissue and blood myeloid cells (high expression in yellow tone). (C) Volcano plots representing significant miRNA gene loci from tissue vs blood comparison in dendritic cells and macrophages. Top miRNA loci are indicated on the plot. FC correspond to differences in detection rate.

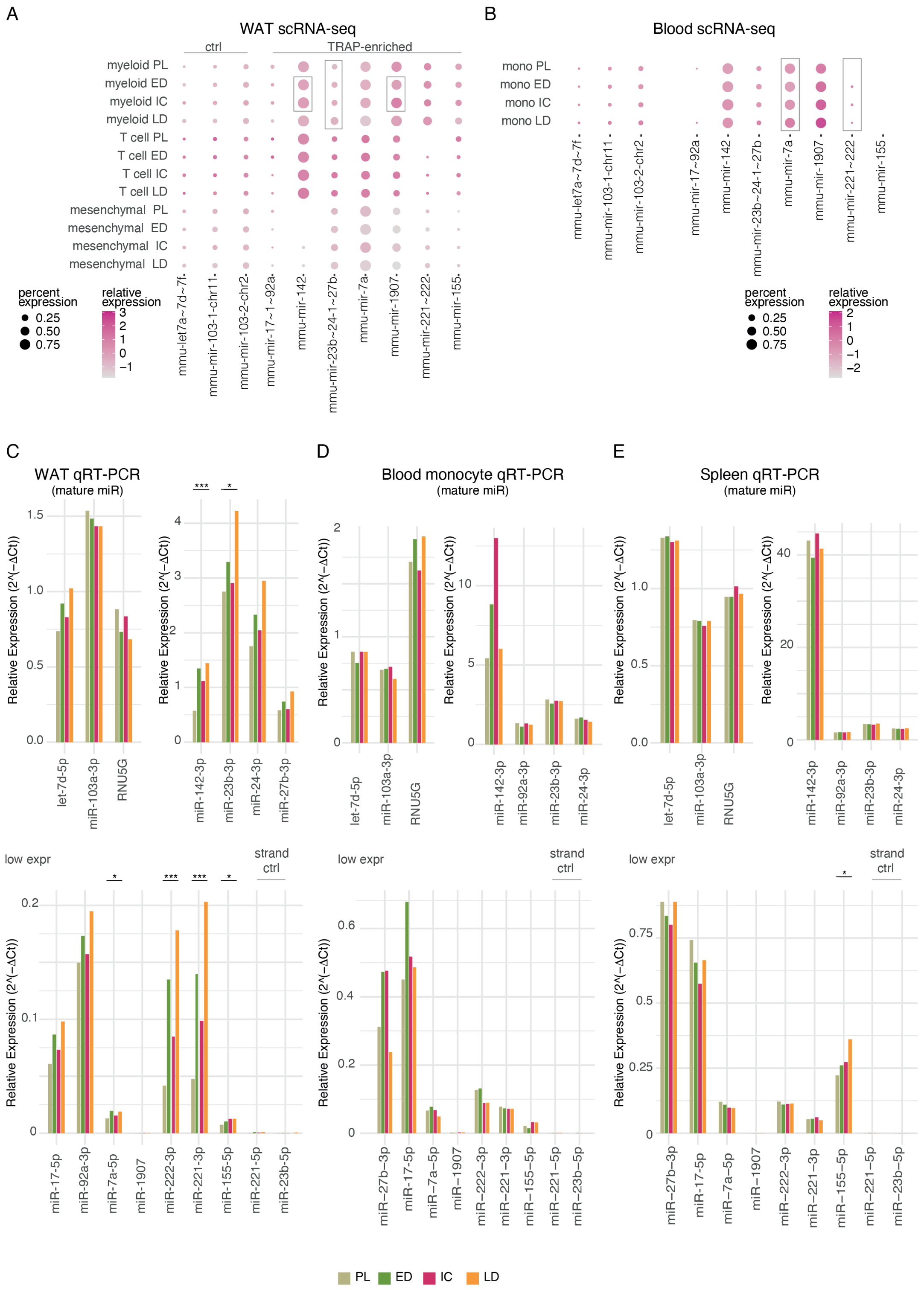

Fig. S6. Comparison of miRNA gene and mature miRNA expression in WAT and blood monocytes. Expression of miRNAs selected for analysis in WAT (A) and blood (B) scRNA-seq data by cell type and sample group is shown as dotplot. (C-E) Expression level of mature miR normalized by the three control genes shown (top left) for highly expressed miR (top right) and low expressed miR (bottom) in WAT (C, n=6 except LD n=3), blood monocytes (D, data from pool of 3 animals) and spleen (E, n=6 per group). Statistical significance based on Student's t-test for PL vs. LD comparison is indicated by stars (* p-value < 0.01; ** p-value < 0.001; *** p-value < 0.0001). Refer to Additional file 6: Table S5 for full statistical summary. PL: pre-lesioned, ED: early disease, IC: inflammatory challenge, LD: late disease.
